# A Next Generation of Hierarchical Bayesian Analyses of Hybrid Zones Enables Model‐Based Quantification of Variation in Introgression in R

**DOI:** 10.1002/ece3.70548

**Published:** 2024-11-21

**Authors:** Zachariah Gompert, Devon A. DeRaad, C. Alex Buerkle

**Affiliations:** ^1^ Department of Biology Utah State University Logan Utah USA; ^2^ Department of Ecology & Evolutionary Biology University of Kansas Lawrence Kansas USA; ^3^ Department of Botany University of Wyoming Laramie Wyoming USA

**Keywords:** admixture, Bayesian inference, genomic clines, hybrid zone, R package

## Abstract

Hybrid zones, where genetically distinct groups of organisms meet and interbreed, offer valuable insights into the nature of species and speciation. Here, we present a new R package, bgchm, for population genomic analyses of hybrid zones. This R package extends and updates the existing bgc software and combines Bayesian analyses of hierarchical genomic clines with Bayesian methods for estimating hybrid indexes, interpopulation ancestry proportions, and geographic clines. Compared to existing software, bgchm offers enhanced efficiency through Hamiltonian Monte Carlo sampling and the ability to work with genotype likelihoods combined with a hierarchical Bayesian approach, enabling inference for diverse types of genetic data sets. The package also facilitates the quantification of introgression patterns across genomes, which is crucial for understanding reproductive isolation and speciation genetics. We first describe the models underlying bgchm and then provide an overview of the R package and illustrate its use through the analysis of simulated and empirical data sets. We show that bgchm generates accurate estimates of model parameters under a variety of conditions, especially when the genetic loci analyzed are highly ancestry informative. This includes relatively robust estimates of genome‐wide variability in clines, which has not been the focus of previous models and methods. We also illustrate how both selection and genetic drift contribute to variability in introgression among loci and how additional information can be used to help distinguish these contributions. We conclude by describing the promises and limitations of bgchm, comparing bgchm to other software for genomic cline analyses, and identifying areas for fruitful future development.

## Graphical abstract

The paper introduces a new R package, bgchm, designed for population genomic analyses of hybrid zones, offering efficient hierarchical Bayesian methods to estimate genetic ancestry and patterns of introgression across genomes. It extends existing software by integrating Hamiltonian Monte Carlo sampling and hierarchical Bayesian approaches. The package is demonstrated through the analysis of simulated and empirical data.

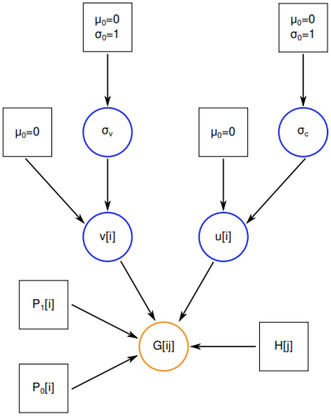

## 1 Introduction

Hybrid zones form when genetically distinct groups of organisms meet, mate, and produce offspring (Barton and Hewitt 1985; Gompert and Buerkle 2016). Studies of hybrid zones provide powerful opportunities to analyze interactions between divergent gene pools in the wild (Barton, Gale, and Harrison 1993; Buerkle and Lexer 2008; Gompert, Mandeville, and Buerkle 2017) and are especially relevant for testing hypotheses about the nature and genetic basis of species and speciation (Harrison and Larson 2014; Firneno et al. 2023). The ease with which genomic data can be generated has vastly increased the potential for genomic analyses of hybrid zones. Simultaneously, advances in analytical approaches and computer software packages have increased the ability of investigators to make evolutionary inferences from hybrid zone data (reviewed in Gompert, Mandeville, and Buerkle 2017).

Hybrid zone theory was largely developed in the mid to late 1900s (e.g., Haldane 1948; Endler 1977; Barton 1979, 1983; Barton and Hewitt 1985). Results from this body of theory provide a means to connect model parameters describing the width, location, and shape of geographic clines in hybrid zones to evolutionary parameters and processes, such as selection and dispersal (Barton and Hewitt 1985). Such geographic cline approaches have been used extensively and productively in speciation research (e.g., Szymura and Barton 1986; Mallet et al. 1990; Dasmahapatra et al. 2002; Carling and Brumfield 2008; Teeter et al. 2008; Westram et al. 2021; Caeiro‐Dias et al. 2023). Nonetheless, these approaches are not always applicable, especially when hybridization lacks a major geographic axis (e.g., Harrison and Rand 1989; Rieseberg, Whitton, and Gardner 1999; Mandeville et al. 2015; Chaturvedi et al. 2020) and are but one of the several windows into the evolutionary processes provided by hybrid zones.

The prevalence and genomic composition of hybrids in hybrid zones provides additional information about the strength and form of reproductive isolation (Jiggins and Mallet 2000). Moreover, genomic approaches can go beyond simple classification of hybrids as F1s, F2s, or backcrosses by describing hybrid genomes quantitatively. For example, genome composition can be measured with a hybrid index, which denotes the proportion of an individual's genome inherited from one of two designated hybridizing lineages (Buerkle 2005), and by an interpopulation (i.e., interclass) ancestry proportion, which indicates the proportion of an individual's genome with gene copies inherited from both hybridizing species (Gompert and Buerkle 2010; Fitzpatrick 2012; Gompert et al. 2014; Shastry et al. 2021). Admixture proportions summarize genome composition similarly to hybrid indexes but without specifying reference or source populations (Pritchard, Stephens, and Donnelly 2000). Together these metrics provide flexible, continuous summaries of the genetic makeup of hybrids that are relevant for understanding hybrid zone dynamics. For example, interpopulation ancestry will be high when matings between non‐admixed individuals or between hybrids and non‐admixed individuals are common.

Additionally, genomic cline models can be used to quantify introgression from one genomic background to another, with a focus on patterns of heterogeneity in introgression across the genome and associated evolutionary processes (Szymura and Barton 1986; Gompert and Buerkle 2011; Macholán et al. 2011; Fitzpatrick, 2013b). In this context, recombination and independent assortment in hybrids create new genotypic combinations that are subject to selection based on their effects on hybrid fitness. Such selection, along with other factors (e.g., patterns of linkage disequilibrium) and processes (e.g., recombination, drift, gene flow, etc.), affect patterns of introgression in hybrid zones (Barton 1983; Gompert, Parchman, and Buerkle 2012; Lindtke and Buerkle 2015; Schumer et al. 2018; McFarlane et al. 2021). Consequently, outcomes of hybridization and patterns of introgression often vary across the genome (e.g., Nolte, Gompert, and Buerkle 2009; Larson et al. 2013; Sung et al. 2018; Chaturvedi et al. 2020; Wagner et al. 2020; Caeiro‐Dias et al. 2023), which can provide additional information about the genetics of speciation (Payseur 2010; Harrison and Larson 2016; Gompert, Mandeville, and Buerkle 2017). This variation can be quantified using genomic cline models and compared across sets of SNPs, genetic regions, chromosomes, and hybrid zones with implications for understanding the genetics of reproductive isolation, the repeatability of speciation, and coupling of barrier loci in hybrid zones (e.g., Teeter et al. 2010; Larson et al. 2013; Taylor et al. 2014; Nikolakis et al. 2022; Firneno et al. 2023; McFarlane et al. 2023).

Here, we present a new R package, bgchm, which combines Bayesian analyses of hierarchical genomic clines with Bayesian methods for (i) estimating hybrid indexes and interpopulation ancestry proportions and (ii) fitting geographic cline models. This package builds on the foundation of the existing bgc software (Gompert and Buerkle 2012) but replaces the Barton cline model with the logit‐logistic cline model proposed by Fitzpatrick (2013b). We describe the details of the models and software usage below but here briefly highlight some of the most salient aspects of this R package (we make detailed comparisons with related software in the Discussion). First, bgchm replaces traditional random‐walk Metropolis‐Hastings Markov chain Monte Carlo with Hamiltonian Monte Carlo, which generally results in less autocorrelation among samples from posterior distributions (Neal 2011). As with the original bgc, bgchm retains the ability to analyze data comprising known genotypes or to work directly with genotype likelihoods, which are the standard output of most modern genetic variant callers and imputation methods. This makes it possible to account for uncertainty in genotypes in analyses and is critical for accurate and powerful inference from low to moderate coverage DNA sequence data sets. bgchm additionally adds the option to work directly with local (locus‐specific) ancestry estimates instead of genotypic data. Finally, bgchm retains a hierarchical Bayesian approach to cline inference. Together, these features result in more reliable inference of cline standard deviation parameters, which provide model‐based summaries of variation in introgression across the genome and are relevant for studying coupling in hybrid zones (Firneno et al. 2023). Additionally, by separately estimating hybrid indexes and clines, but still retaining the hierarchical structure of the model, bgchm drastically improves parallelization relative to bgc and also allows comparisons among different sets of loci (e.g., trait associated versus putatively neutral loci, or different chromosomes) without assuming all loci in a set share the same cline parameters. These features are important for scaling cline analyses to genome‐level data sets, with limitations mostly set by the availability of CPUs.

In this manuscript, we first describe the core models underlying bgchm. We then provide an overview of the R package and illustrate its use through the analysis of simulated data sets, with a focus on the effects of hierarchical modeling and allele frequency differences between reference populations, and on our ability to estimate cline standard deviations. Where relevant, we compare bgchm to HIest (Fitzpatrick 2013a), which provided the original implementation of the logit‐logistic cline model. We further demonstrate the usage of bgchm via the analysis of a butterfly hybrid zone data set. We conclude by discussing the potential and limitations of bgchm, comparing this R package with other hybrid zone analysis software and identifying possibilities for further developments.

## 2 Methods

### 2.1 Models

We consider three sets of models to describe genomic patterns of admixture and introgression in hybrid zones, specifically, models to infer hybrid indexes, ancestry class proportions, and genomic clines (geographic cline models are described in the Supporting Information). All three models infer ancestry from defined source or reference populations and use a (hierarchical) Bayesian approach for inference and quantifying uncertainty in model parameters. We first describe these models for the case where genotypes are assumed to be known without error before presenting extensions for modeling genotype uncertainty or working directly with local ancestry estimates.

#### 2.1.1 Hybrid Index Model

We follow the basic structure of the hybrid index model proposed by Buerkle (2005). Here, hybrid indexes are defined with respect to two putative source or reference populations chosen to represent or approximate the genetic composition of two hybridizing species or lineages. The hybrid index for individual, j, $H_{j}$, denotes the proportion of individual j's genome that is best modeled as being inherited from one of the two source populations (labeled source 1). Consequently, $1-H_{j}$ denotes the proportion of the genome inherited from the other source population (labeled source 0). Hybrid indexes are based on supervised learning of allele frequencies within source populations that are defined *a priori* and are analogous to admixture proportions estimated in an unsupervised learning context (Pritchard, Stephens, and Donnelly 2000; Gompert et al. 2014). Here, we consider only two source populations. We assume that the genotypic data for individual j and locus i is binomially distributed conditional on ancestry of the alleles at locus i and the corresponding parental allele frequencies ($P_{0i}$ and $P_{1i}$) and, similarly, that the ancestry at locus i is binomially distributed conditional on the hybrid index, $H_{j}$. This results in the following likelihood model for estimating hybrid indexes:

(1) $$\begin{array}{c} \mathrm{Pr}\left(G_{\mathrm{ij}}|H_{\mathrm{ij}},P_{0i},P_{1i}\right)\propto {\left(H_{\mathrm{ij}}P_{1i}+\left(1-H_{\mathrm{ij}}\right)P_{0i}\right)}^{G_{\mathrm{ij}}}\\ {\left(H_{\mathrm{ij}}\left(1-P_{1i}\right)+\left(1-H_{\mathrm{ij}}\right)\left(1-P_{0i}\right)\right)}^{\left(N_{\mathrm{ij}}-G_{\mathrm{ij}}\right)} \end{array}$$

Here, $G_{\mathrm{ij}}$ is the count of one of the two alleles (e.g., the non‐reference allele), and $N_{\mathrm{ij}}$ denotes the number of allele copies for the individual and locus (i.e., two for diploids). At present, we restrict analysis to diploid or haploid loci, including mixtures of diploid and haploid loci as might occur with sex chromosomes. We assume that any allele with a non‐zero frequency in the putative hybrids has a non‐zero frequency in at least one of the source populations. The information about ancestry (and thus hybrid index) a locus provides depends on the allele frequency difference between the reference populations and thus varies from a maximum for loci with fixed differences, to no information, in cases where the allele frequencies are identical. Missing data can be accommodated by the model and does not contribute to the hybrid index estimate for an individual. We assume a beta prior on hybrid indexes, such that $H_{j}∼\text{beta}\left(\alpha =0.5,\beta =0.5\right)$, which corresponds with Jeffreys minimally informative prior.

#### 2.1.2 Ancestry Class Proportions Model

Our model for ancestry class proportions is similar to the interpopulation ancestry models described by Gompert et al. (2014) and Shastry et al. (2021) (i.e., the Q model). However, unlike these models, our ancestry class proportion model assumes that source populations are defined with known allele frequencies a priori (i.e., supervised learning, as in our hybrid index model). We designate the ancestry class proportions $Q_{00}$, $Q_{11}$, and $Q_{10}$ to denote (i) the proportion of an individual's genome where both gene copies were inherited from source (i.e., reference) population 0 ($Q_{00}$), (ii) the proportion of an individual's genome where both gene copies were inherited from source population 1 ($Q_{11}$), and (iii) the proportion of an individual's genome where one gene copy was inherited from each source population ($Q_{10}$, i.e., interpopulation ancestry). The main purpose of the model is to estimate these ancestry class proportions. Note that hybrid index can be derived directly from the ancestry class proportions as $H=Q_{11}+\frac{1}{2}Q_{10}$. We define a likelihood analogous to Equation (1) for the ancestry class proportions as:

(2) $$\;\mathrm{Pr}\left(G_{\mathrm{ij}}|Q_{j},P_{0i},P_{1i}\right)\propto \left\{\begin{array}{ll} Q_{11j}P_{1i}^{2}+Q_{00j}P_{0i}^{2}+Q_{01j}P_{1i}P_{0i} & \text{if}\;G_{\mathrm{ij}}=2\\ Q_{11j}\left(1-P_{1i}\right)P_{1i}+Q_{00j}\left(1-P_{0i}\right)P_{0i}+ & \\ Q_{01j}\left(1-P_{1i}\right)P_{0i}+Q_{01j}P_{1i}\left(1-P_{0i}\right) & \text{if}\;G_{\mathrm{ij}}=1\\ Q_{11j}{\left(1-P_{1i}\right)}^{2}+Q_{00j}{\left(1-P_{0i}\right)}^{2}+ & \\ \;Q_{01j}\left(1-P_{1i}\right)\left(1-P_{0i}\right) & \text{if}\;G_{\mathrm{ij}}=0 \end{array}\right.$$

Here also, we restrict the analysis to haploid and diploid loci. Haploid loci provide information about the proportion of the genome inherited from each species but not how this is partitioned into homozygous versus heterozygous (interpopulation) ancestry, the latter comes only from the diploid loci.

#### 2.1.3 Genomic Clines Model

Genomic clines represent the probability of locus‐specific ancestry along a genome‐wide admixture gradient, that is, as a function of hybrid index (Szymura and Barton 1986; Gompert and Buerkle 2009, 2011). Here, we model genomic clines with the logit‐logistic model proposed by Fitzpatrick (2013b), which was derived from a sigmoidal geographic cline model (this does not imply a sigmoidal genomic cline). With this function, the probability that a gene copy for locus i and individual j was inherited from source population 1 (as opposed to source population 0) is $\phi_{\mathrm{ij}}=H_{j}^{v_{i}}/\left(H_{j}^{v_{i}}+\left(1-H_{j}^{v_{i}}\right)e^{u_{i}}\right)$, where $H_{j}$ is the hybrid index (i.e., the proportion of the genome inherited from population 1), $v_{i}$ gives the slope of the cline for locus i relative to the genome‐average ($\bar{v}=1$), and $u_{i}$ specifies the center of the cline for locus i relative to both the genome average and $v_{i}$ (Fitzpatrick 2013b). We use the re‐parameterization from Bailey (2024) and Firneno et al. (2023) that defines $\text{logit}\left(c_{i}\right)=\frac{u_{i}}{v_{i}}$ to specify a more intuitive cline center parameter ($c_{i}$), which indicates the hybrid index value at which $\phi_{\mathrm{ij}}$ = 0.5, that is where the probability of inheriting an allele from each source population is equal. Genomic cline slopes greater than 1 indicate a steeper cline than the admixture gradient, whereas clines less than 1 indicate a shallower cline. Similarly, centers greater than 0.5 indicate an overall excess of source 0 ancestry, whereas centers less than 0.5 indicate an excess of source 1 ancestry.

We specify the following likelihood model for the genetic data at locus i given in terms of $\phi_{\mathrm{ij}}$, which is itself a function of hybrid index ($H_{j}$, a property of an individual) and the cline parameters $v_{i}$ and $c_{i}$ (properties of a locus):

(3) $$\begin{array}{c} \mathrm{Pr}\left(G_{\mathrm{ij}}|\phi_{\mathrm{ij}},P_{0i},P_{1i}\right)\propto {\left(\phi_{\mathrm{ij}}P_{1i}+\left(1-\phi_{\mathrm{ij}}\right)P_{0i}\right)}^{G_{\mathrm{ij}}}\\ \;{\left(\phi_{\mathrm{ij}}\left(1-P_{1i}\right)+\left(1-\phi_{\mathrm{ij}}\right)\left(1-P_{0i}\right)\right)}^{\left(N_{\mathrm{ij}}-G_{\mathrm{ij}}\right)}. \end{array}$$

Note the similarity between Equation (3) and Equation (1); the forms are identical except that $\phi_{\mathrm{ij}}$ (the probability of ancestry from source 1 for locus i and individual j) in Equation (3) replaces $H_{j}$ (the marginal probability of ancestry from source 1 for individual j) in Equation (1). Here also, we allow for diploid and haploid loci as well as loci with mixed ploidy (e.g., sex chromosomes).

**FIGURE 1:**
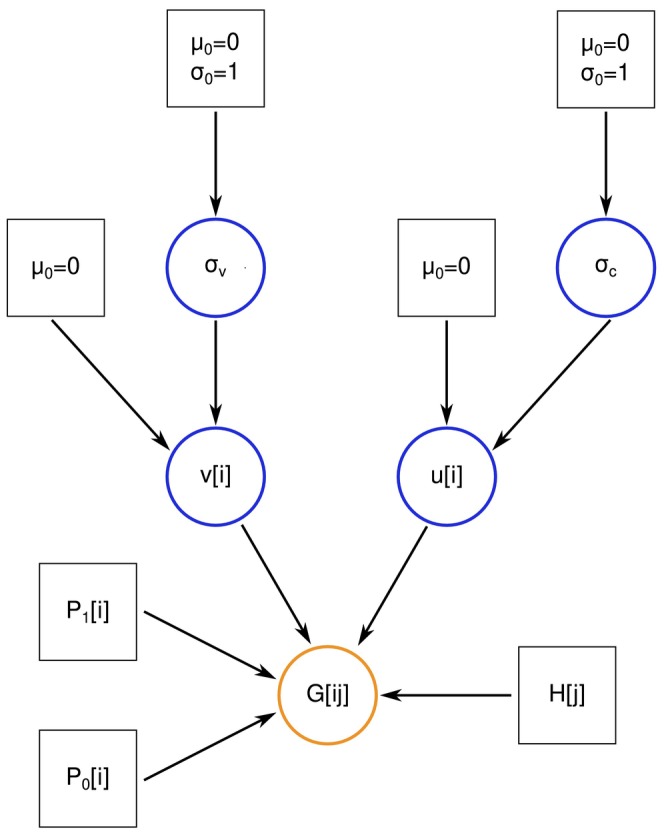
Directed graph summarizing the (standard) hierarchical Bayesian genomic cline model. Boxes and circles denote fixed and stochastic nodes, respectively, with the data node in orange and other stochastic nodes in blue. $G\left[i,j\right]$ denotes the genetic data for locus i and individual j, that is, the known genotype or genotype likelihoods. $P_{0}\left[i\right]$ and $P_{1}\left[i\right]$ are the known (previously estimated) allele frequencies in parental source or reference populations. $H\left[j\right]$ is the known (previously estimated) hybrid index for individual j. The stochastic nodes $v\left[i\right]$ and $u\left[i\right]$ are the cline parameters, with $v\left[i\right]$ denoting the slope and $u\left[i\right]=\text{logit}\left(\text{center}\left[i\right]\right)v\left[i\right]$, where $\text{center}\left[i\right]$ is the cline center. $\sigma_{v}$ and $\sigma_{c}$ denote the standard deviations of the normal priors on $\log\left(v\left[i\right]\right)$ and $\text{logit}\left(\text{center}\left[i\right]\right)$. These describe variability in clines across the genome and are estimated from the data. The remaining fixed nodes denote means ($\mu_{0}$) and standard deviations ($\sigma_{0}$) of higher‐level normal priors.

Following Gompert and Buerkle (2011) and Firneno et al. (2023), we define hierarchical priors for the cline parameters $v_{i}$ and $c_{i}$. Hierarchical modeling allows information on the genomic variability of introgression to be shared across loci and explicitly acknowledges the partial dependence (and partial independence) of introgression across the genome (Gompert and Buerkle 2011; Betancourt and Girolami 2015) (Figure 1). In general, hierarchical modeling in such cases is conceptually preferable to the alternative assumptions of complete independence of units (e.g., introgression patterns across loci) as implied by fixed, independent priors, or the complete lack of independence among units as implied by a shared parameter for all units (e.g., the same cline parameters for all loci; Gelman et al. 1995; Fordyce et al. 2011). In our case, the main benefit of hierarchical modeling is the ability to learn about and account for the degree of variability in clines among loci. In our standard model, we specify the following priors for cline parameters (modifications are discussed below): $\log_{10}\left(v_{i}\right)∼\text{normal}\left(\mu =0,\sigma =\sigma_{v}\right)$ and $\text{logit}\left(c_{i}\right)∼\text{normal}\left(\mu =0,\sigma =\sigma_{c}\right)$. The log and logit functions are used to set the expected means of $v_{i}$ and $c_{i}$ to 0– $\log_{10}\left(1\right)=0$ and $\text{logit}\left(0.5\right)=0$–and also to project these parameters onto the scale of −∞ and ∞. The means of both priors are set to 0 to reflect the fact that, assuming the same loci (or random subsets of the same loci) are used to infer hybrid indexes and to fit genomic clines, the average deviation of locus‐specific clines from the genome average should by definition be 0 (Gompert and Buerkle 2011). This can be relaxed in cases where distinct sets of loci are used for cline fitting and hybrid indexes, as we discuss below. Such a zero‐centered prior does not enforce a hard sum‐to‐zero constraint but rather serves as a form of soft centering. We discuss hard‐centering (i.e., sum‐to‐zero constraints) in Section 2.2.

The standard deviation parameters, $\sigma_{v}$ and $\sigma_{c}$ describe the variability of cline slopes and centers across the genome and can be related to the extent of coupling among loci (Barton 1983; Firneno et al. 2023). Specifically, with coupling, linkage disequilibrium among loci causes selection arising from one locus to indirectly result in selection on other loci such that the loci experience similar levels of selection and exhibit concordant and coincident clines (as well as steeper geographic clines). This should manifest as lower levels of variation in genomic clines across the loci with the extreme case being clines for each locus coinciding precisely with the genome‐average cline ($v_{i}=1$ and $c_{i}=0.5$). The standard deviation parameters simultaneously inform and are informed by the locus‐specific cline parameters, and it is this co‐dependency that allows information sharing across loci. As such, these cline standard deviations ($\sigma_{v}$ and $\sigma_{c}$) are estimated from the data as part of the analysis (at least in the standard model, modifications to this procedure are discussed below). Thus, priors (hyperpriors) are placed on the standard deviations, $\sigma_{v}∼\text{normal}\left(\mu =0,\sigma =\sigma_{0}\right)$ and $\sigma_{c}∼\text{normal}\left(\mu =0,\sigma =\sigma_{0}\right)$, with $\sigma_{0}$ set by users. Note that this specification assumes that the loci are exchangeable, which is likely not true for sets of tightly linked loci. However, the cline standard deviations can be inferred from a subset of unlinked or loosely linked loci.

#### 2.1.4 Alternative Model Specifications and Assumptions

Having described a standard version of each of our models for hybrid indexes, ancestry class proportions, and genomic clines above, we now discuss modifications and variants of these models. First, the model descriptions above assume that genotypes are known without error (or completely missing). However, modern sequencing technologies and bioinformatic tools generate finite numbers of reads or sequences covering each segment of DNA, uncertain base calls, and mapping errors. These sources of uncertainty mean that genotypes are often uncertain. This is reflected in the genotype likelihoods output by most variant calling software (e.g., samtools and bcftools; Li 2011). Uncertainty can also arise from genotype imputation or Bayesian inference of genotypes (in these cases, uncertainty is often captured in a posterior probability rather than a likelihood but can be incorporated in the same manner). Thus, we include modifications of all three core models to incorporate uncertainty in genotypes (including equal likelihoods of all genotypes) by working directly with relative likelihoods or posterior probabilities of genotypes (e.g., as output by some Bayesian genotype inference methods, e.g., Shastry et al. 2021). In such cases, the likelihoods given in Equations ((1), (2), (3)) are replaced by the average likelihood of the parameter values conditional on each genotype and weighted by relative genotype likelihoods or posterior probabilities. For example, Equation (3) becomes $\begin{array}{c} \mathrm{Pr}\left(G_{\mathrm{ij}}|\phi_{\mathrm{ij}},P_{0i},P_{1i}\right)=\mathrm{Pr}\left(G_{\mathrm{ij}}=0|\phi_{\mathrm{ij}},P_{0i},P_{1i}\right)\mathrm{Pr}\left(G_{\mathrm{ij}}=0\right) \end{array}$ $\begin{array}{c} +\mathrm{Pr}\left(G_{\mathrm{ij}}=1|\phi_{\mathrm{ij}},P_{0i},P_{1i}\right)\mathrm{Pr}\left(G_{\mathrm{ij}}=1\right)+\mathrm{Pr}\left(G_{\mathrm{ij}}=2|\phi_{\mathrm{ij}},P_{0i},P_{1i}\right)\mathrm{Pr}\left(G_{\mathrm{ij}}=2\right) \end{array}$

We also consider alternative models where we assume that locus‐specific ancestry is itself known or has been estimated using one of many programs designed for local‐ancestry inference (e.g., Li and Stephens 2003; Maples et al. 2013; Browning, Waples, and Browning 2023). With known local ancestry, the likelihood equations no longer depend on parental allele frequencies, and, for example, Equation (3) can be simplified to (this is mathematically equivalent to a model for diagnostic markers):

(4) $$\mathrm{Pr}\left(Z_{\mathrm{ij}}|\phi_{\mathrm{ij}}\right)\propto \phi_{\mathrm{ij}}^{Z_{\mathrm{ij}}}{\left(1-\phi_{\mathrm{ij}}\right)}^{N_{\mathrm{ij}}-Z_{\mathrm{ij}}}$$

Here, $Z_{\mathrm{ij}}$ denotes the ancestry of locus i in individual j, that is, the number of gene copies (out of $N_{\mathrm{ij}}$) individual j inherited from source population 1 at this specific locus. Local ancestry can be defined for, and inferred from, individual SNPs or larger loci, such as haplotype blocks.

We define two additional variants of the genomic clines model, both of which can be applied with known genotypes, uncertain genotypes, or local ancestry. First, one such variant allows the standard deviations of the hierarchical priors, $\sigma_{v}$ and $\sigma_{c}$, to be specified and fixed. As described in more detail in Section 2.2, this makes it possible to first estimate these parameters using the standard hierarchical model based on a random subset of data and then to fix these parameters for estimating clines for the full set of data, enabling massive parallelization of the model fitting procedure across genetic loci. Alternatively, this model formulation can be used to specify weakly informative priors (i.e., relatively flat priors) and thereby implement a non‐hierarchical version of the genomic clines model akin to Bailey (2024).

Second, in some cases, it can be useful to estimate the means of the hierarchical priors for v and c from the data rather than fix them at 0. As we illustrate in an example analysis below, this could be done if one estimates hybrid indexes based on one subset of loci (e.g., putative neutral regions of the genome, autosomes only, etc.) and then wants to ask whether a different subset of loci (e.g., trait‐associated loci, other candidate genes, and sex‐linked loci, etc.) exhibit patterns of introgression that deviate on average from the subset of loci used for hybrid index inference. Consequently, we have also included models with unknown means for the hierarchical priors, $\log_{10}\left(v_{i}\right)∼\text{normal}\left(\mu =\mu_{v},\sigma =\sigma_{v}\right)$ and $\text{logit}\left(c_{i}\right)∼\text{normal}\left(\mu =\mu_{c},\sigma =\sigma_{c}\right)$. In such cases, we place normal priors on the unknown means as well as the unknown standard deviation, with $\mu_{v}∼\text{normal}\left(\mu =0,\sigma =\mu_{0}\right)$ and $\mu_{c}∼\text{normal}\left(\mu =0,\sigma =\mu_{0}\right)$, and with the prior standard deviation for these means, $\mu_{0}$, specified by the user.

### 2.2 Software Usage

We implemented the models described above in a new R package, bgchm, which updates the original bgc program (Gompert and Buerkle 2012). The R package is available for direct installation from GitHub at https://github.com/zgompert/bgc‐hm. The R package uses Stan (via rstan) for sampling from posteriors (Stan Development Team 2022, 2024). This implementation makes it possible to fit the models using Hamiltonian Monte Carlo (HMC) rather than using more traditional Markov chain Monte Carlo algorithms. This is important as HMC routinely outperforms other algorithms especially in terms of convergence and more effectively exploring complex posterior distributions (Neal 2011; Betancourt and Girolami 2015). This means that far fewer HMC steps are generally required to obtain a good approximation of the posterior distribution and that the HMC algorithm is less likely to get stuck in one region of the posterior, especially when fitting hierarchical models and estimating higher‐level standard deviations (Betancourt and Girolami 2015). We specifically use the No‐U‐Turn Sampler (NUTS) from Stan (Hoffman, Gelman, et al. 2014; Betancourt 2017). Integration with Stan and rstan also provides built‐in diagnostics of HMC performance, including automated and standard warning messages about performance and estimates of effective sample sizes and convergence diagnostics for each of the model parameters. Moreover, by using Stan, all of the HMC sampling is done based on compiled C++ code, rather than native R code, which is critical for reducing the time required for model fitting.

The R package bgchm includes core functions for estimating hybrid indexes (est_hi), ancestry class proportions (est_Q), and genomic clines (est_gencline). Each function is documented in the R package. The arguments to these functions determine which version of each model to fit, with each version corresponding to an internal compiled C++ program. The result of any Bayesian analysis is the full posterior distribution for the set of model parameters. Samples from this distribution are provided with each of the core functions as well as useful summaries of the central tendency (median) of the posterior and uncertainty in estimates (credible intervals). Separate helper functions exist for estimating parental allele frequencies (est_p) (this can also be done within the three core functions), summarizing posterior distributions (pp_plot), and visualizing results (e.g., producing triangle interpopulation ancestry plots or plotting genomic clines). Additional functions for hierarchical geographic cline analyses are described in the Supporting Information (these are not the main focus of the software but are included for the convenience of users interested in hierarchical geographic cline models). We assume that reasonable filtering and quality control of the genetic data have been completed prior to analysis with bgchm.

We have made the core functions modular for flexibility and ease of scaling though this comes at the cost of not fully propagating uncertainty in parental allele frequencies and hybrid indexes in genomic cline analyses (this is an unfortunate but somewhat necessary trade‐off). Consequently, the parental allele frequencies required for hybrid index, ancestry class proportions, and genomic clines can be estimated within bgchm or provided from other software. Likewise, the hybrid indexes used in the genomic cline analysis can be estimated for all or a subset of loci and can be inferred within bgchm or using other software (e.g., these could be admixture proportions from a model with k=2 in structure; Pritchard, Stephens, and Donnelly 2000). This set‐up allows for extensive parallelization of cline fitting and thus makes it possible to run bgchm on genome‐scale data sets as long as one has access to sufficient computational resources. Specifically, a standard analysis can begin by estimating hybrid indexes based on a moderate number of loci; several hundred to a thousand will generally be sufficient to obtain precise estimates of hybrid indexes. Then, for modest sized data sets (up to a few thousand loci and a few hundred hybrids), the full set of loci can be analyzed in a single hierarchical model (the standard model described above). For larger data sets (more than a thousand individuals or loci, and up to millions of SNPs), a subset of hundreds or a few thousand (unlinked or loosely linked) loci can be fit in an initial hierarchical model to estimate the cline standard deviation parameters, $\sigma_{v}$ and $\sigma_{c}$. These parameters can then be fixed at their point estimates and the clines for the remaining loci can be fit in batches (and thus in parallel across CPUs or computer nodes) using these estimated standard deviations (runtimes for individual SNPs are on the order of a few seconds). This gains most of the benefit of using a hierarchical modeling framework without the cost of needing to fit clines for a very large number of loci in a single model. Additional parallelization is possible for all analyses by running multiple HMC chains in parallel (this is done within the bgchm program). We provide an example of batch parallelization of loci in the bgchm repository (https://github.com/zgompert/bgc‐hm), including a UNIX shell script to control the batch parallelization, and we have successfully used this approach to fit clines for > 1 million SNPs in about 2 days on a single compute node with 48 CPUs.

As noted above in Section 2.1, the hierarchical prior structure for the standard genomic cline model results in soft centering of the cline parameters, such that the mean of the cline parameters (on the appropriate log or logit scale) is shrunk towards zero. However, this is not the same as a hard, sum‐to‐zero constraint, as implemented in the original bgc program (Gompert and Buerkle 2012), which forces the mean of the cline parameters to be zero. We found that trying to enforce a hard sum‐to‐zero constraint within the HMC algorithm dramatically degraded performance of the algorithm. Moreover, a hard sum‐to‐zero constraint would only be possible when fitting all loci in a single model. We have thus instead opted to use soft centering, while also providing a function, sum2zero, that applies a sum‐to‐zero constraint to a set of cline parameter estimates after model fitting. This can be done based on the full HMC output (preferable when practical) or simply as an adjustment to the parameter estimates (useful when saving the full HCM output for all loci is computationally burdensome). Either of these options can be applied after batch processing of cline estimation for many loci and thus makes it possible to apply a sum‐to‐zero constraint to genome‐scale data. We think this is advisable in most cases, at least when the loci used to estimate hybrid indexes are the same or a random subset of those used to estimate clines. We think this because the genomic clines are, by definition, deviation from average introgression. In some cases, the soft centering might be sufficient to effectively constrain the mean of cline parameters to zero such that applying the hard sum‐to‐zero constraint is not necessary, but in other cases, including in the example empirical analysis we present, this is not true (this will depend—in ways that are yet to be fully investigated—on the distribution of hybrid indexes, variability of clines among loci, and other aspects of the data set and posterior distribution).

### 2.3 Analyses of Simulated Data Sets

We analyzed a series of simulated data sets to illustrate and evaluate the performance of the bgchm package. Aspects of this or related models have been analyzed extensively elsewhere and thus are not treated in depth here. For example, Gompert, Parchman, and Buerkle (2012) evaluated the concordance between loci with exceptional genomic cline parameters (from the original bgc model) and loci causally affecting fitness (this varies depending on the genetic architecture of fitness variation). Firneno et al. (2023) used simulations to assess the relationship between Barton's theoretical coupling coefficient (θ) and the cline standard deviations from the hierarchical Bayesian logit‐logistic model described here. Firneno et al. (2023) then quantified these cline standard deviations across a series of empirical data sets. Bailey (2024) examined the sensitivity of a non‐hierarchical implementation of the logit‐logistic cline model to the distribution of hybrid indexes. Here, our main focus is on demonstrating the general performance of the software and exploring specific aspects of these models or performance that have received less attention, including the effects of hierarchical modeling, allele frequency differences between parents, and our ability to accurately estimate cline standard deviations.

#### 2.3.1 Analyses of Simulations of Hybrid Indexes and Ancestry Class Proportions

We first conducted simulations to evaluate the ability of bgchm to accurately estimate hybrid indexes and ancestry class proportion from genetic data. Simulations were conducted using dfuse under a model of neutral secondary contact (Lindtke and Buerkle 2015). The program dfuse implements individual‐based simulations to model a hybrid zone that forms following secondary contact. The program tracks hybrid indexes, ancestry class proportions (specifically our $Q_{10}$), and ancestry junctions along chromosomes. As such, it provides a way to simulate hybrids where the core parameters for these models, H and Q, are known. We conducted 50 replicate simulations of 200 generations where hybridization occurs in a single admixed deme with an adult carrying capacity of 500. The migration rate from the parental populations to the deme was set to 0.1. We simulated hermaphroditic, diploid organisms with ten chromosomes, each one Morgan in length. We output ancestry information for 51 loci spaced evenly along each of the ten chromosomes (510 loci total). At the end of each simulation, we randomly sampled 50 individuals from the hybrid zone deme for analysis. We then generated three genotypic data sets based on the output from each replicate simulation. Specifically, we sampled genotypes for each individual and locus based on the individual's local ancestry and assumed parental allele frequencies of (i) 0 and 1, (ii) 0.25 and 0.75, (iii) or 0.45 and 0.55 for parents 0 and 1, respectively. This corresponds with parental allele frequency differences of 1 (fixed differences), 0.5 and 0.1. Genotypes were generated using binomial sampling (in R). From each of these genotypic data sets, we created an additional data set where the genotypes were uncertain. For this, we assumed the number of sequence reads for each individual and locus followed a Poisson distribution with λ = 7 and these sequences had a 1% error rate (the inherent per‐base pair error rate for Illumina sequences is ∼0.31%, Schirmer et al. 2016). Reads were sampled in R based on the genotypes and these parameters, and the likelihood of each genotype was then computed from the reads assuming the 1% error rate. Thus, for each of the 50 initial simulated hybrid zones, we generated six genetic data sets: parental allele frequency differences of 1, 0.5, or 0.1 with genotypes known or uncertain.

We then estimated hybrid indexes and ancestry class proportions with bgchm using the est_hi and est_Q functions. We did this using the model for known genotypes or genotype likelihoods as appropriate and with default HMC conditions for these functions: four HMC chains with 2000 steps, including 1000 warmup iterations. We used the known parental allele frequencies for the analysis. We summarized the posterior estimates of hybrid index and ancestry class proportions for each individual and simulated data set based on the posterior median (point estimate) and 90% credible intervals (CIs, specifically the 90% equal‐tail probability intervals). We then evaluated performance by computing the mean absolute error (MAE) and the proportion of 90% CIs containing the true parameter value (90% CI coverage) for each data set.

#### 2.3.2 Genomic Cline Analyses of Simulated Hybrid Zones

We next conducted a series of simulations and analyses to evaluate the performance of the genomic cline models in bgchm. The first two sets of simulations were designed to evaluate the conditions under which bgchm could accurately estimate genomic cline parameters. Unlike hybrid indexes and ancestry class proportions, individual‐based simulations, such as those in dfuse, do not generate known cline parameters. Thus, for these sets of simulations, we instead simulated hybrids using the logit‐logistic genomic cline model as a generative model. The first set of simulations was designed to evaluate the effect of cline variability, that is variability in introgression across the genome, on our ability to accurately estimate cline parameters. For this, we considered three levels of cline variability: low ($\sigma_{v}$ = 0.2 and $\sigma_{c}$ = 0.5), moderate ($\sigma_{v}$ = 0.4 and $\sigma_{c}$ = 0.8), and high ($\sigma_{v}$ = 0.6 and $\sigma_{c}$ = 1.2) (for context, compare these to estimates of the same parameters across a series of empirical data sets in Firneno et al. 2023). We simulated 50 data sets for each level of cline variability. In each case, we sampled the cline parameters v and c from normal distributions (on the $\log_{10}$ and logit scale, respectively) with means of zero and standard deviations of $\sigma_{v}$ and $\sigma_{c}$. Cline parameters were sampled for 100 loci per data set. We then sampled hybrid indexes for 50 hybrids per data set; these were drawn from a uniform distribution bounded by 0 and 1. We then computed the locus‐specific ancestry for each locus i and individual j based on the cline parameters and hybrid index, $\phi_{\mathrm{ij}}=H_{j}^{v_{i}}/\left(H_{j}^{v_{i}}+\left(1-H_{j}^{v_{i}}\right)e^{u_{i}}\right)$, with $u_{i}=\text{logit}\left(c_{i}\right)v_{i}$. Local ancestry states for each locus and individual ($Z_{\mathrm{ij}}$) were then sampled from a binomial distribution with two draws using $\phi_{\mathrm{ij}}$ as the probability of ancestry from source population 1. In these initial simulations, we assumed fixed differences between source populations, such that ancestry was fully informative of state.

We then estimated cline parameters for each of the 150 data sets (50 replicates with each of three cline standard deviations). We analyzed the data using the standard hierarchical Bayesian genomic cline model in bgchm and with two alternative models: (i) a non‐hierarchical variant of the genomic cline model in bgchm with the prior cline standard deviation set to be relatively uninformative ($\sigma_{c}$ and $\sigma_{v}$ = 100) and the corresponding logit‐logistic genomic cline model in HIest (version 2.0; Fitzpatrick 2013a). The comparison with the non‐hierarchical model was done to evaluate the effect of modeling the clines hierarchically versus not doing so. The comparison with HIest was chosen as this was the initial software developed to fit this form of genomic cine model (with a non‐hierarchical model) and thus serves as a general check on the quality of our inference. Notably, only the hierarchical model provides a means to estimate the cline standard deviations and HIest requires fixed differences between parents (hence our focus on loci with fixed differences for this initial set of simulations). Genomic clines in HIest were fit using the L‐BFGS‐B algorithm. Models fit with bgchm used default HMC settings of 2000 iterations, including a 1000 iteration warmup, and no thinning. Four chains were run. For the hierarchical models, the priors on the standard deviations for $\sigma_{v}$ and $\sigma_{c}$ were normal with means of 0 and standard deviations of $\sigma_{0}$ = 2. We used the known parental allele frequencies and hybrid indexes for all analyses (with the caveat, the parental allele frequencies of 0.001 and 0.999 were used rather than 0 and 1 to avoid problems with infinite probabilities during computation).

We conducted a second set of simulations to evaluate the effects of allele frequency differences between source populations and uncertainty in genotypes on the ability of bgchm to estimate genomic cline parameters. For this, we again simulated data using the logit‐logistic genomic cline model as a generative model. Here, we considered only a case of intermediate variability in introgression across the genome, that is, $\sigma_{v}$ 0.3 = and $\sigma_{c}=$ 0.7 (this is between the low and moderate variability cases considered for the first set of simulations). We simulated three levels of allele frequency differences between source populations: (i) fixed differences, (ii) SNPs with a minimum allele frequency difference of 0.5, and (iii) SNPs with a minimum allele frequency difference of 0.1. In each case, actual allele frequency differences for each SNP were sampled from a uniform distribution bounded by 1 and the specified lower bound (e.g., 0.5 or 0.1). Thus, allele frequency differences varied among loci (except in the case of all fixed differences), as would be expected for many empirical data sets. We simulated 50 data sets comprising 100 loci and 50 hybrids for each level of minimum allele frequency differences. Then, for each simulation, we generated an additional, complementary data set with uncertain genotypes. This was done as described above for the hybrid index and ancestry class proportion analyses. Specifically, we again assumed a Poisson distributed number of reads per individual and locus (λ = 7) and 1% sequence error rate.

Next, we estimated genomic cline parameters for each of the 300 simulated data sets (50 replicates for each level of allele frequency differences and for genotypes known versus uncertain) using the hierarchical model from bgchm. We did not include the comparison with HIest as this program requires diagnostic allele frequency differences between source populations and we kept our focus on the hierarchical model to evaluate inferences of cline standard deviations. We used the default HMC settings of 2000 iterations, including a 1000 iteration warmup, and no thinning. Four chains were run. Priors on the standard deviations for $\sigma_{v}$ and $\sigma_{c}$ were normal with means of 0 and standard deviations of $\sigma_{0}$ = 2. We again used the known parental allele frequencies and hybrid indexes for all analyses.

#### 2.3.3 Genomic Cline Analyses of Hybrid Zones Simulated With Dfuse

We then conducted a third set of simulations to examine the relationship between the genetic architecture of hybrid fitness and cline parameters, including both clines for individual loci and the cline parameter standard deviations. These simulations were not meant to be exhaustive but rather to complement existing simulation‐based studies of genomic clines in the context of the genetics of isolation in hybrids and cline coupling (e.g., Gompert, Parchman, and Buerkle 2012; Firneno et al. 2023). Our purpose was to illustrate how different genetic architectures of hybrid fitness can leave different patterns in genomic clines and how these relate to patterns that might arise in the absence of selection.

Hybrid zones were simulated using dfuse (Lindtke and Buerkle 2015). We described this software and model previously in the context of the simulations used to assess our hybrid index and ancestry class proportion models. In these individual‐based simulations, cline parameters are not strictly defined–it is not guaranteed that the patterns of introgression will conform precisely to the form specified by the genomic cline model nor are the parameters of such a model defined by the simulation conditions. Thus, we do not use these simulations to assess the accuracy of the bgcmh model per se but rather to evaluate how cline parameter estimates are affected by the simulation conditions. Here, we assumed that hybrid fitness is determined by N underdominant loci, such that the fitness of an individual heterozygous for ancestry at n of the N loci is $w_{j}={\left(1-s\right)}^{n}$, where s is the selection coefficient (the underdominance model was added to dfuse in Firneno et al. (2023). We simulated ten replicate data sets under four different hybrid zone models. All simulations involved secondary contact, 15 demes for the hybrid zone, an adult carrying capacity of 100 individuals per deme, a migration rate of 0.05 between neighboring demes, and 5000 generations of evolution post secondary contact. We simulated hermaphroditic, diploid organisms each with one, 1 Morgan chromosome. We recorded ancestry at 251 evenly spaced loci along the chromosome of each individual. One set of simulations involved no selection (i.e., neutral evolution by drift and gene flow only). A second set assumed an oligogenic architecture of fitness with two underdominant loci with s = 0.3 at positions 25 cM and 75 cM along the chromosome (an individual heterozygous at both loci would have a relative fitness of 0.49). The third set of simulations considered a polygenic architecture with weak selection overall, specifically 50 underdominant loci distributed at even distances across the chromosome and with s = 0.005 per locus (an individual heterozygous at all 50 loci would have a relative fitness of 0.78). The last set of simulations was of strong polygenic selection, which again involved 50 evenly distributed underdominant loci but with s = 0.01 (an individual heterozygous at all 50 loci would have a relative fitness of 0.61).

We randomly sampled 100 individuals from each simulated hybrid zone for analysis. We assumed fixed differences between source populations at the 251 loci, such that ancestry was perfectly informative of genotype. Genomic cline parameters were estimated using the standard hierarchical model in bgchm. We used the known hybrid indexes and parental allele frequencies of 0.001 and 0.999. We again used the default HMC settings of four chains each comprising 2000 iterations including a 1000 iteration warmup and no thinning. We set normal priors for $\sigma_{v}$ and $\sigma_{c}$ with means of 0 and standard deviations of $\sigma_{0}$ = 2.

### 2.4 Analysis of an Example Empirical Data Set

Lastly, to demonstrate possible usages of the genomic cline models in bgchm, we applied them to an empirical genetic data set from a hybrid zone in *Lycaeides* butterflies. The data set was originally published and analyzed in Chaturvedi et al.'s (2020). Two nominal species of *Lycaeides* butterflies, *L. idas* and *L. melissa* , occur throughout much of western North America with partially overlapping ranges (Nabokov 1943; Gompert et al. 2010, 2014). These species differ on average in terms of the structure of the male genitalia (Nabokov 1944; Gompert et al. 2010), aspects of wing pattern (Lucas, Nice, and Gompert 2018), host plant species used, and voltinism (Gompert et al. 2013) but nonetheless have hybridized extensively (Gompert et al. 2010, 2012a; Nice et al. 2013; Gompert et al. 2014; Chaturvedi et al. 2020). An ancient, partially stabilized series of admixed populations occurs in the central Rocky mountains and Jackson Hole, which we refer to as Jackson Hole *Lycaeides*. These populations are the product of hybridization between *L. idas* and *L. melissa* that occurred about 14,000 years ago following the retreat of Pleistocene glaciers. More recently, Jackson Hole *Lycaeides* have come into secondary contact with *L. melissa* near the town of Dubois, WY (43.5623°N, 109.6991°W) where *L. melissa* feed on naturalized alfalfa ( *Medicago sativa* ) that grows along roadsides and that was introduced to North America about 250 years ago. This recent secondary contact has resulted in a contemporary hybrid zone (Chaturvedi et al. 2020; Zhang et al. 2023), which is the focus of our analyses here. Our goals here are to use bgchm to characterize the genomic composition of this hybrid zone in terms of hybrid indexes, ancestry class proportions, and genomic cline parameters. We then specifically examine the extent to which clines differ on average between autosomes and the Z sex chromosome and as a function of features of the genome (gene and transposable element density).

We focus on a data set comprising the Dubois hybrid zone (N = 115) individuals, three populations representative of source Jackson Hole *Lycaeides* (set as source 0, N = 166), and two populations representative of source *L. melissa* populations (set as source 1, N = 117) (see Figure S1). We identified ancestry informative loci from a larger set of 39,193 SNPs generated from genotyping‐by‐sequencing data (see Chaturvedi et al. 2020 for details, including variant filtering and genotype inference). We specifically considered ancestry‐informative SNPs, here defined as those with an allele frequency difference of 0.3 or greater between our source populations; this yielded a total of 500 ancestry informative SNPs (330 such SNPs on the 22 autosomes and 170 on the Z chromosome). We began by estimating hybrid indexes and ancestry class proportions based on this full data set with the est_hi and est_Q functions in bgchm. This was done using the known genotype model with maximum likelihood estimates of parental allele frequencies derived from Bayesian point estimates of genotypes. We treated Z‐linked SNPs in females as haploid. We used the default HMC conditions for these functions, that is four HMC chains with 2000 steps, including 1000 warmup iterations and no thinning. We summarized the posterior estimates of hybrid index and ancestry class proportions for each individual based on posterior medians and 90% CIs.

We next fit several genomic cline models to illustrate different ways cline estimates can be used to make inferences. First, as the primary analysis, we fit a single hierarchical genomic cline model using all 500 SNPs. Here, we used the hybrid indexes estimated from the full data set, maximum likelihood estimates of parental allele frequencies, and again treated Z‐linked SNPs as haploid in females. We fit the model with the default HMC conditions–four chains each comprising 2000 iterations including a 1000 iteration warmup and no thinning–with the prior mean for the cline standard deviations set to 0 and the prior standard deviations set to 2 (i.e., $\sigma_{c}$ and $\sigma_{v}$ were estimated from the data). We applied the sum‐to‐zero constraint to cline estimates from this analysis.

We then fit an additional pair of genomic cline models to directly ask whether patterns of introgression differed on average for autosomes versus the Z chromosome. For this, we used hybrid indexes estimated only from the autosomes. Genomic cline parameters for the autosomes and Z chromosome were then estimated separately, that is, in separate fits of genomic cline models. Here, not only did we estimate the cline standard deviations from the data ($\sigma_{c}$ and $\sigma_{v}$) but also the mean ($\mu_{c}$ and $\mu_{v}$). Because the hybrid indexes were based on the autosomal data, the expected means for the autosomal SNPs were $\mu_{c}$ = 0 and $\mu_{v}$ = 0. However, this was not true for the Z chromosome SNPs and the values of $\mu_{c}$ and $\mu_{v}$ for these SNPs thus indicate the extent and manner in which patterns of introgression deviate on average for Z‐linked SNPs versus autosomes. With that said, the values of $\mu_{c}$ and $\mu_{v}$ for autosomes are not forced to be 0, and thus, we based our inferences on the difference in $\mu_{c}$ and $\mu_{v}$ for Z for autosome SNPs (specifically, on the posterior distribution for such differences). These models were also fit the default HMC conditions, but with normal priors on $\mu_{c}$ and $\mu_{v}$, both with means of 0 and standard deviations of $\sigma_{0}$ = 2.

We conducted a final set of cline model fits to explicitly compare the variability of clines across autosomes versus the Z chromosome relative to the average introgression on autosomes versus the Z chromosome. For this, we estimated hybrid indexes separately for autosomal and Z loci; we then fit hierarchical cline models for these sets of loci separately using the autosomal and Z‐based hybrid indexes, respectively. We fixed cline means to 0 (as per the standard model) and estimated the cline standard deviations, $\sigma_{c}$ and $\sigma_{v}$, which were the main focus of this analysis. This was again done with the standard HMC settings with the standard deviation of the normal prior on the cline standard deviations set to $\sigma_{0}$ = 2. For all analyses we summarized the posterior estimates of cline parameters (v and c), hierarchical cline standard deviations, and hierarchical cline means based on posterior medians and 90% CIs.

## 3 Results

### 3.1 Analyses of Simulated Data Sets

#### 3.1.1 Results for Simulations of Hybrid Indexes and Ancestry Class Proportions

**FIGURE 2:**
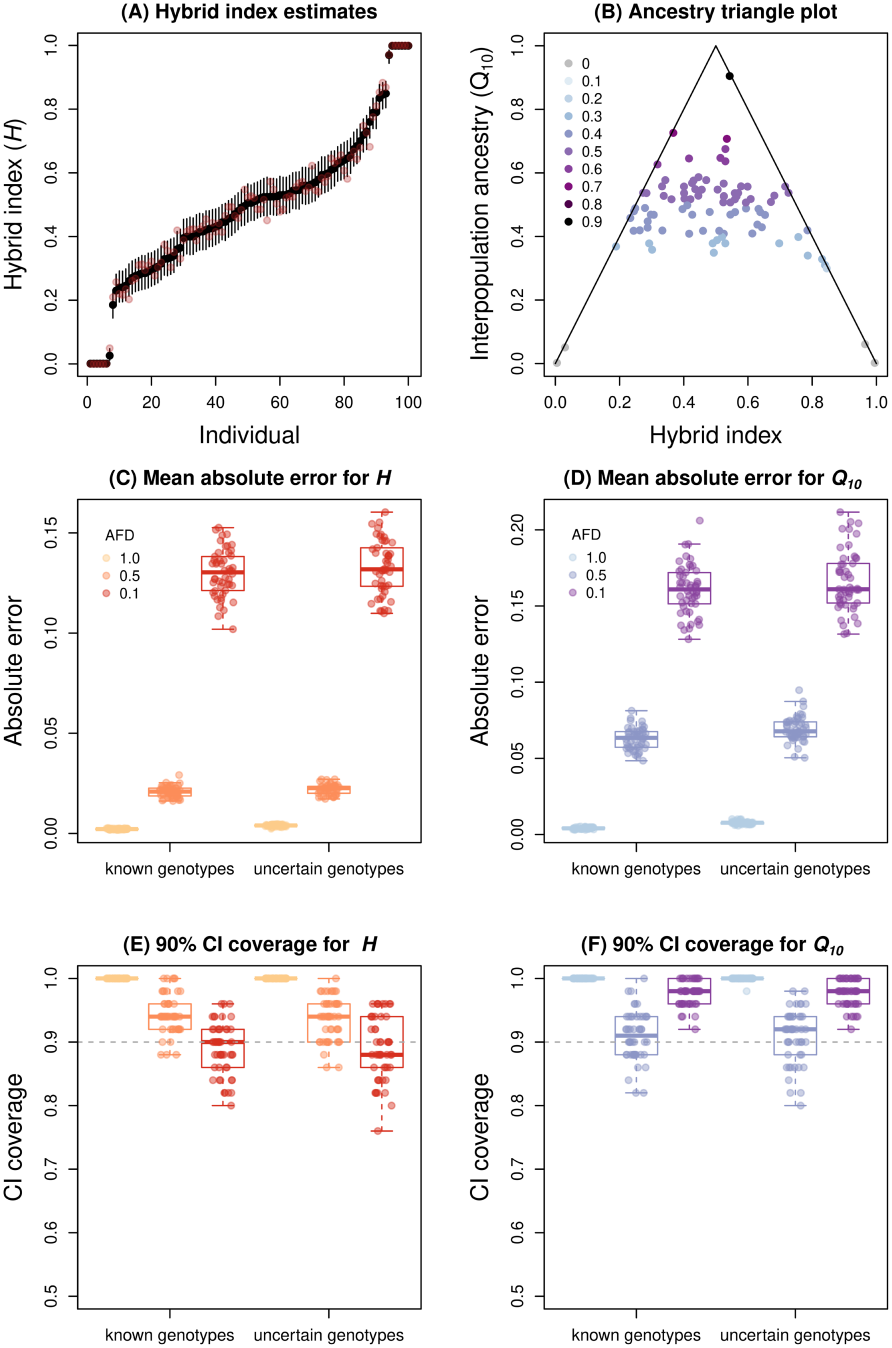
Example results and summary evaluation of model performance for estimating hybrid index (H) and interpopulation ancestry ($Q_{10}$). Panel (A) shows point estimates of hybrid index (black points) and 90% credible intervals (CIs) (vertical lines) for 100 individuals. This is based on 100 loci with fixed differences between parental populations. Pink points show the true, simulated hybrid index values. The triangle plot in panel (B) shows interpopulation ancestry estimates ($Q_{10}$) as a function of hybrid index (H) for the same simulated individuals. Point colors indicate true parameter values (in increments of 0.1) and lines (the triangle) denote maximum values of interpopualtion ancestry for a given hybrid index. Points on or near this line denote likely offspring with one non‐hybrid parent. Panels (C)–(F) summarize model performance for 50 replicate simulations each with allele frequency differences (AFDs) between parents of 1.0, 0.5, or 0.1 and known or uncertain genotypes. Panels (C) and (D) summarize mean absolute error for estimates of H and $Q_{10}$, respectively. Boxes indicate the median and 1st and 3rd quartiles of the distribution across replicate simulations, with whiskers extending up to 1.5 × the interquartile range. The overlain points show metrics for individual replicates. Panels (E) and (F) similarly summarize the proportion of loci where the true parameter value is within the 90% CI of the Bayesian estimate for H (E) and $Q_{10}$ (F). The horizontal dashed line denotes the expectation of 90% for a 90% CI.

Example graphical summaries of hybrid index (H) and interpopulation ancestry ($Q_{10}$) estimates are shown in Figure 2A,B. In general, performance was slightly better for hybrid index than interpopulation ancestry (Table S1 and Figure 2). Mean absolute error (MAE, the average deviation between true and estimated parameter values) increased with decreasing allele frequency differences, that is, with reduced information on ancestry in the genotypic data (Figure 2). However, even with allele frequency differences of 0.1, average MAEs were below 0.14 for H and 0.17 for $Q_{10}$ (Table S1). Moreover, 90% credible intervals generally contained the true parameter value 90% of the time or more. Indeed, for the simulations with the greatest allele frequency differences (i.e., 1; fixed differences), the CIs appear to be conservative, with the true values of H and $Q_{10}$ almost always falling within the 90% CIs. Inferences based on appropriately modeled uncertain genotypes were nearly as accurate as those based on known genotypes (Table S1 and Figure 2).

#### 3.1.2 Results From Genomic Cline Analyses of Simulated Hybrid Zones

**FIGURE 3:**
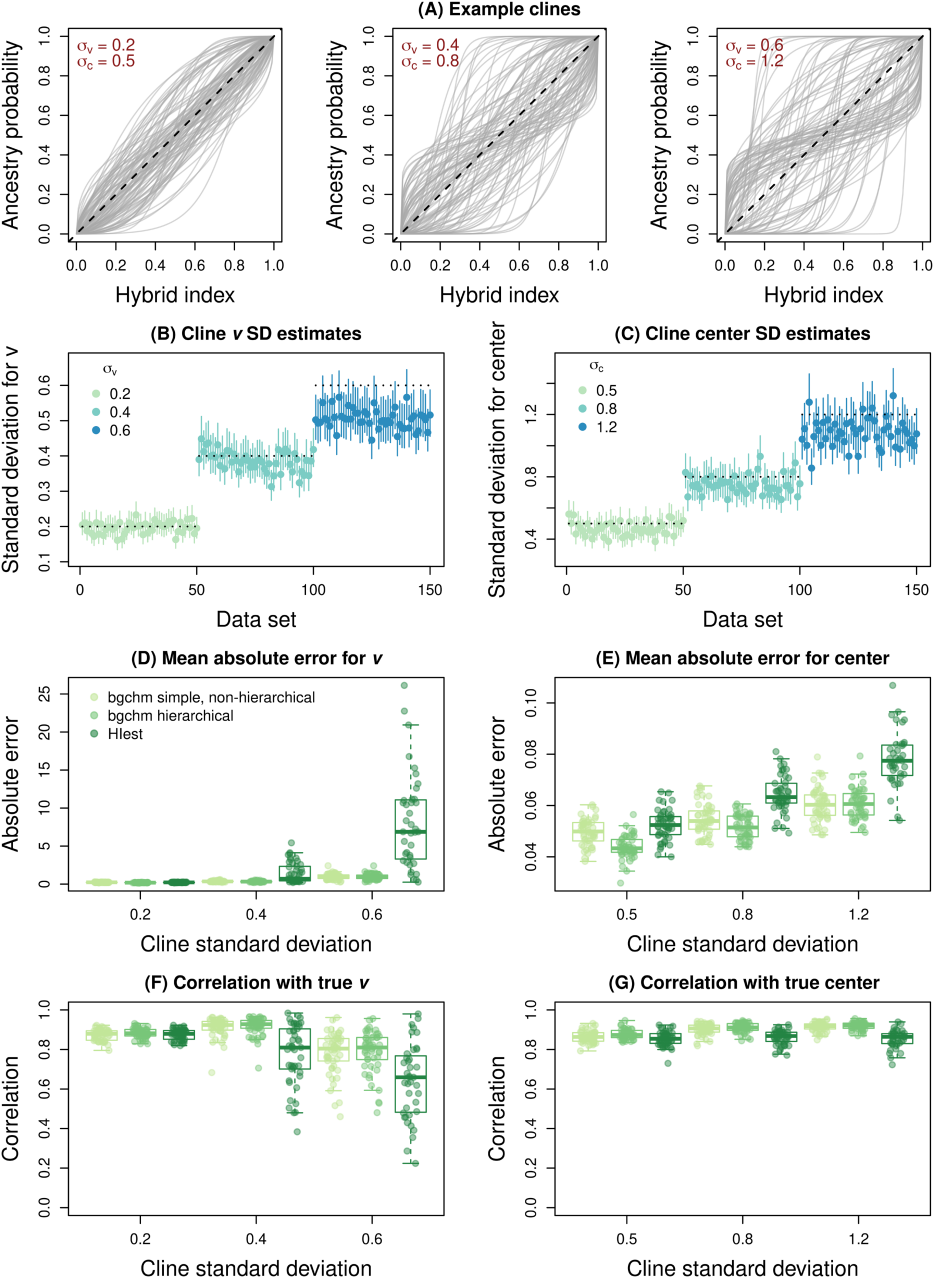
Summary of genomic cline variability and the effect of such variability on cline inference. Panel (A) shows simulated genomic clines with low ($\sigma_{v}$ = 0.2 and $\sigma_{c}$ = 0.5), moderate ($\sigma_{v}$ = 0.4 and $\sigma_{c}$ = 0.8), and high ($\sigma_{v}$ = 0.6 and $\sigma_{c}$ = 1.2) variability in introgression. Each gray line is the cline for a locus and gives the probability of ancestry from source 1 as a function of hybrid index (the overall proportion of the genome from source 1). The null expectation if introgression does not vary across the genome is given by the dashed black line. Estimates of cline standard deviations for slope, $\sigma_{v}$, and center, $\sigma_{c}$, are shown in panels (B) and (C), respectively. Here, point estimates and 90% credible intervals (CIs) are depicted with points and vertical lines. Horizontal dotted lines give the true value used for each simulation. Performance, in terms of estimating cline slopes (v) and centers, is summarized based on mean absolute error in panels (D) and (E) and in terms of the correlation between true and estimated parameter values in panels (F) and (G). Errors and Pearson correlations were computed based on parameter point estimates (posterior medians) and are summarized across replicate simulations with boxplots. Boxes indicate the median and 1st and 3rd quartiles of the distribution across replicate simulations, with whiskers extending up to 1.5 × the interquartile range. The overlain points show metrics for individual replicates. Performance of bgchm using a simple non‐hierarchical model and a hierarchical model are shown, as are results from HIest (for HIest, cases where the algorithm failed are excluded).

Example clines for loci with fixed differences and with low ($\sigma_{v}$ = 0.2 and $\sigma_{c}$ = 0.5), moderate ($\sigma_{v}$ = 0.4 and $\sigma_{c}$ = 0.8), and high ($\sigma_{v}$ = 0.6 and $\sigma_{c}$ = 1.2) variability in introgression across the genome are shown in Figure 3A. Under these conditions, estimated cline standard deviations were highly correlated with the true cline standard deviations, with Pearson correlations of 0.97 (95% confidence interval = 0.96–0.98) and 0.97 (95% confidence interval = 0.95–0.98) for $\sigma_{v}$ and $\sigma_{c}$, respectively (Figure 3B,C). With that said, when cline variability was high, variation was somewhat underestimated, such that the mean estimates of $\sigma_{v}$ and $\sigma_{c}$ for the highest variability case were 0.50 and 1.09 compared to the true values of 0.6 and 1.2. Such a bias was not apparent for the low variability simulations (mean $\sigma_{v}$ = 0.20 and mean $\sigma_{c}$ = 0.47, compared to true values of 0.2 and 0.5).

With regard to individual cline parameters, MAE was generally higher when clines were more variable, and likewise, correlations between true and estimated values declined (more so for slope than center) (Table S2, Figure 3). In general, bgchm outperformed HIest, especially when cline variability was high. The hierarchical and non‐hierarchical models performed similarly, but with slightly better performance in terms of error and correlations with true parameter values for the hierarchical model, especially when cline variance was low (Table S2). As expected, our results also suggest that, relative to the non‐hierarchical model with weakly informative priors, the hierarchical model is conservative in the sense that it induces some shrinkage towards zero into the parameter estimates (see Table S3). Furthermore, $\sigma_{v}$ and $\sigma_{c}$ can only be estimated as model parameters in the hierarchical model.

**FIGURE 4:**
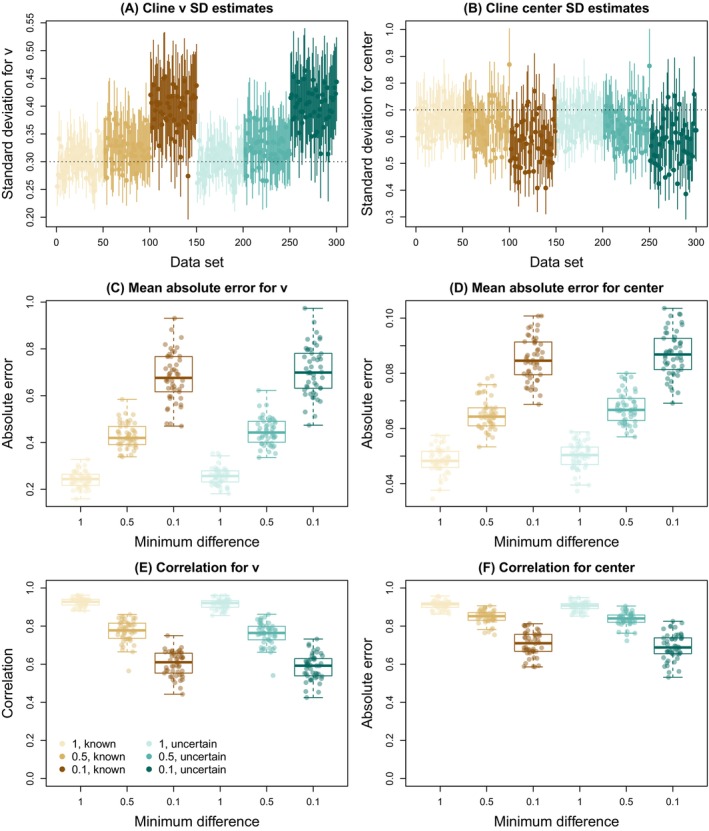
Summary of the effects of source allele frequency differences and genotype uncertainty on genomic cline inference. All panels show results based on minimum source allele frequency differences of 1, 0.5 and 0.1 and with or without uncertainty in genotypes as indicated by the colors and associated legend. Panels (A) and (B) provide estimates of cline standard deviations for slope, $\sigma_{v}$, and center, $\sigma_{c}$, respectively. Points and vertical lines depict point estimates and 90% credible intervals (CIs) Horizontal dotted lines give the true value used for $\sigma_{v}$ (A) and $\sigma_{c}$ (B). Model performance for genomic cline parameters (v and c) is summarized based on mean absolute error in panels (C) and (D) and based on the correlation between true and estimated parameter values in panels (E) and (F). Errors and Pearson correlations were computed based on parameter point estimates (posterior medians) and are summarized across replicate simulations with boxplots. Boxes indicate the median and 1st and 3rd quartiles of the distribution across replicate simulations, with whiskers extending up to 1.5 × the interquartile range. The overlain points show metrics for individual replicates.

We next considered the effects of source allele frequency differences and genotype uncertainty on estimates of genomic cline parameters with the hierarchical model in bgchm. We found that cline standard deviation estimates ($\sigma_{v}$ and $\sigma_{c}$) were most accurate for fixed differences and became progressively less accurate with low levels of allele frequency differences (Figure 4A,B). When the minimum allele frequency difference between source populations was 0.1, there was a tendency to overestimate the variability in cline slopes (true $\sigma_{v}$ = 0.3, mean point estimate for known genotypes = 0.39) and underestimate the variability in cline centers (true $\sigma_{c}$ = 0.7, mean point estimate for known genotypes = 0.58). Uncertainty in genotypes had little effect on estimates of cline standard deviations (Figure 4A,B). Similarly, cline parameter estimates were most accurate in terms of both MAE and the correlation with true parameter values when allele frequency differences were high and were less accurate when they were low (e.g., 0.1; Table S4, Figure 4). Uncertainty in genotypes tended to further decrease the accuracy of estimates but only to a minor extent (see Table S4, Figure 4). Moreover, the average proportion of loci where the true parameter value was contained in the 90% CIs was only weakly affected by allele frequency differences or genotype uncertainty suggesting that the uncertainty in clines caused by weak genetic differentiation between sources is mostly captured by the uncertainty in parameter estimates (Table S4). With that said, there was slight tendency overall to underestimate cline uncertainty (i.e., between 80% and 88% of the 90% CIs contained the true value relative to the expectation of 90%).

#### 3.1.3 Results From Genomic Cline Analyses of Hybrid Zone Simulated With Dfuse

**FIGURE 5:**
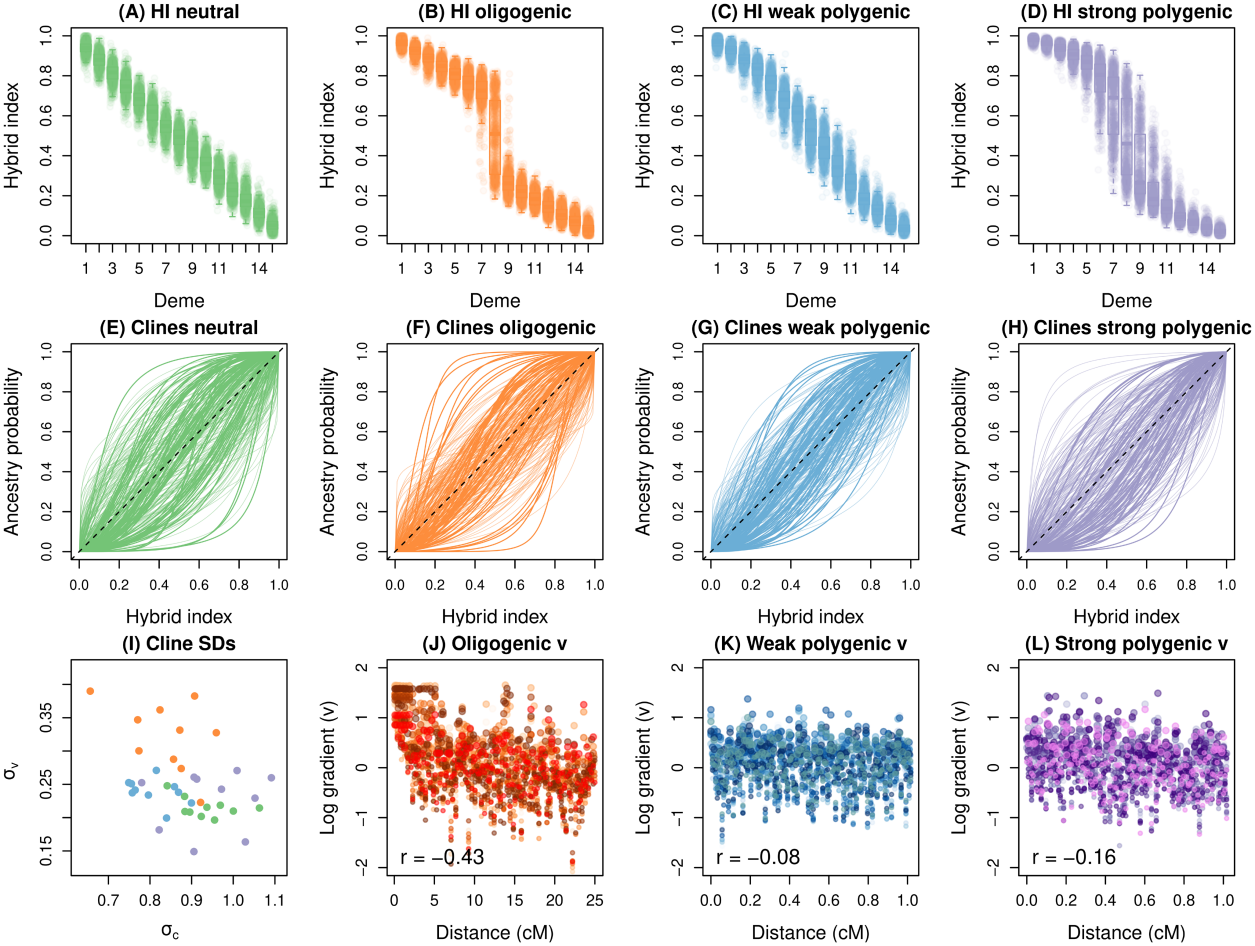
Summary of genomic cline analysis of hybrid zone simulations with alternative genetic architectures for hybrid fitness. Results are shown for neutral secondary contact, oligenic selection, weak polygenic selection and strong polygenic selections (see main text for details). Panels (A–D) show the distribution of hybrid indexes (HI) across demes and across 10 replicate simulations for each set of conditions. Boxes indicate the median and 1st and 3rd quartiles of the hybrid index distribution for each deme, with whiskers extending up to 1.5 × the interquartile range. The overlain points denote individual hybrid indexes. Panels (E–H) show genomic clines from 100 representative loci for each set of conditions. Each colored line is the cline for a locus and gives the probability of ancestry from source 1 as a function of hybrid index. The null expectation if introgression does not vary across the genome is given by the dashed black line. Point estimates of cline standard deviations (SDs) are shown in panel (I). Here, conditions are colored in accordance with panels (A–H). Panels (J), (K) and (L) show the relationships between the distance (in cM) a marker locus is from a selected locus and the log of the cline gradient or slope (v). This is only shown for the three sets of conditions with selection. Points are colored to indicate distinct replicate simulations and the Pearson correlation between distance and $\log\left(v\right)$ is reported.

Our final analysis of simulated hybrid zones involved various genetic architectures for hybrid fitness with dfuse. Overall, stronger selection (oligenic or strong polygenic) resulted in a steeper geographic clines in hybrid indexes across the hybrid zones (Figure 5). However, all four sets of conditions resulted in similar numbers of loci with credible deviations from null expectations for cline slopes (v) and centers (c) (Table S5 and Figure 5). We observed notable variation in cline standard deviations across simulated data sets, with a trend towards larger slope variances ($\sigma_{v}$) for oligogenic selection (Figure 5I).

Despite similar numbers of loci with clines deviating from null expectations, we did find patterns of cline variation consistent with the effects of selection. Specifically, for oligogenic selection and strong polygenic selection, there was a significant (all p<0.05) negative correlation between the log of v and the distance a marker locus was from an underdominant locus (Pearson correlations ranged from −0.34 to −0.49 for oligogenic selection and − 0.11 to −0.23 for strong polygenic selection; Table S6 and Figure 5). Negative correlations were also observed for weak polygenic selection (range = −0.04 to −0.11), but these were not significantly different from 0 (all p>0.05) (Table S6 and Figure 5). No underdominant loci were present in the neutral simulations, thus, as expected, we found small and non‐significant (and mostly positive) correlations between cline slopes and the locations used for underdominant loci in the polygenic simulations (range = −0.02 to 0.06) (Table S6); this demonstrates that large negative correlations do not arise inherently in the absence of selection.

Interestingly, we detected positive correlations between the absolute value of logit cline centers and the location of underdominant loci in the oligogenic simulations and most of the strong polygenic simulations (positive in all ten of the latter, but significantly greater than 0 with p<0.05 for eight of the simulations; Table S7, Figure S2). A similar but non‐significant pattern was documented for weak polygenic selection, and no such pattern was found for neutral simulations (again based on the locations of underdominant loci in polygenic simulations). Thus, at least with stronger selection, simulated SNPs near underdominant loci have steeper cline slopes (larger, positive values of v) but also cline centers closer to the genome‐wide null expectation, suggesting that selection resulted in steeper clines but more constrained (coincident) centers.

### 3.2 Analysis of an Example Empirical Data Set

**FIGURE 6:**
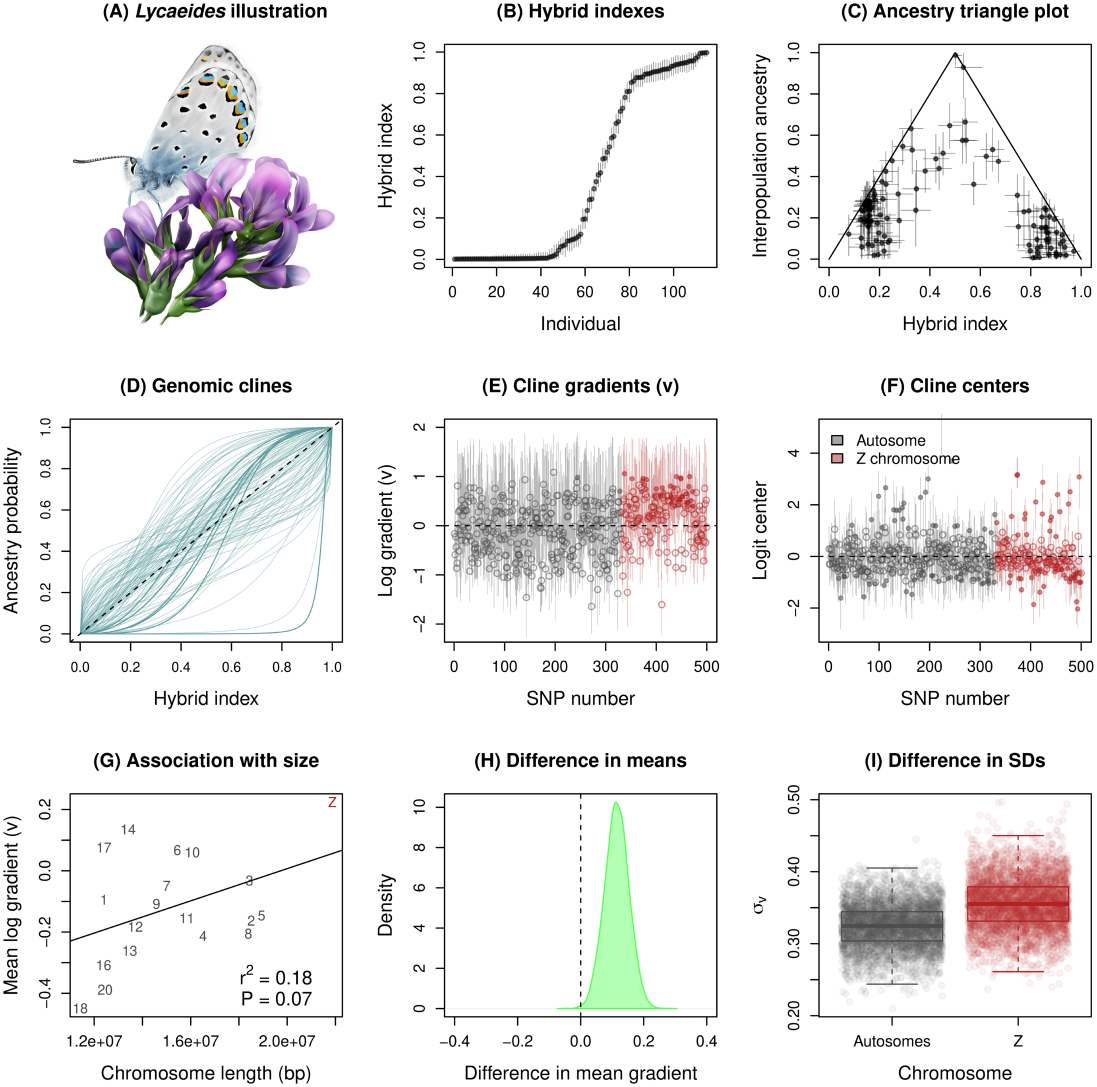
Summary of key results from an example analysis with *Lycaeides* butterflies. An illustration of a *Lycaeides* butterfly from the Dubois hybrid zone is shown in panel (A). Panel (B) gives point estimates (points) and 90% credible intervals (CIs) (vertical lines) for hybrid index based on the combined autosomal and Z chromosome data. Panel (C) shows interpopulation ancestry estimates ($Q_{10}$) as a function of hybrid index (H) for the same hybrid zone butterflies. Point estimates and 90% CIs (vertical and horizontal lines) are given. Genomic clines for 100 representative loci are shown in panel (D). Each line denotes the probability of *L. melissa* ancestry for a locus as a function of hybrid index (the overall proportion of an individual's genome with *L. melissa* ancestry). Darker and thicker lines are used for loci with credible deviations from genome‐average ancestry (90% CIs for cline gradient of center not overlapping null expectations). The diagonal, dashed line shows the null 1:1 expectation for locus‐specific ancestry probabilities as a function of hybrid index. Panels (E) and (F) display estiamtes of the log cline gradient (log of v) and logit cline center for each of the 500 ancestry‐informative SNPs. Point estimates and 90% CIs (vertical lines) are displayed, with open points used for cases where the 90% CIs do not exclude values less than 0 (E) or do not exclude 0 (F). The null expectation value of 0 (on the log or logit scale) is shown for each panel with a horizontal dashed line. Panel (G) shows the relationship between chromosome size (length in base pairs, bps) the the mean log gradient for the 20 chromosomes with more than five ancestry informative SNPs. Chromosome numbers (or Z) are given, along with the best fit line from a linear regression; the model $r^{2}$ and *p*‐value are reported. Panel (H) gives the difference in mean log gradient between the Z chromosome and autosomes for cline models where Z and autosomal SNPs were analyzed separately and where the means were not set to zero but estimated from the data. Both models used autosomal estimates of hybrid indexes. The posterior density for the difference is shown, along with a vertical line for the null expectations. The posterior probability that the mean for Z loci exceeds the mean for autosomes was > 0.99. Panel (I) shows the posterior distributions for the standard deviation in log cline gradients for autosomes and the Z chromosome. Here, autosomal and Z SNPs were analyzed separately and with hybrid indexes inferred from autosomal and Z SNPs, respectively. Boxes indicate the median and 1st and 3rd quartiles of the posterior distribution, with whiskers extending up to 1.5 × the interquartile range. The overlain points show 4000 parameter value samples from the posterior. The posterior probability that the variance for the Z SNPs exceeds the variance for the autosomal SNPs was 0.75.

We estimated hybrid indexes, ancestry class proportions, and genomic cline parameters for 500 ancestry‐informative SNPs in a *Lycaedies* butterfly hybrid zone (Figure 6). Estimates of hybrid indexes were generally precise (mean width of the 90% CIs = 0.061) and spanned the full range from only ancestry from source population 0 (i.e., Jackson Hole *Lycaeides*) to only ancestry from source population 1 (i.e., *L. melissa* ) (Figure 6B). Ancestry class proportion estimates suggest a wide range of genome compositions in hybrids, including some individuals with near maximal interpopulation ancestry for their hybrid indexes (i.e., individuals with one or more non‐admixed parents, that is F1s or backcrosses) and individuals where both parents were likely themselves hybrids (i.e., individuals with lower levels of interpopulation ancestry given their hybrid indexes; Figure 6C).

Genomic cline analysis of all 500 SNPs detected substantial genome‐wide variation in introgression (Figure 6D–F). Overall, patterns of introgression deviated from null expectations based on genome‐average admixture for 218 out of the 500 loci (Table S8). This includes 40 loci with credibly steeper clines than null expectations (v>1), of which 39 were on the Z chromosome. This is a significant enrichment of steep clines on the Z chromosome (randomization test, 1000 randomizations, expected = 13.7, *p* = 0.001). We detected 48 loci with credible excesses in Jackson Hole *Lycaeides* (c>0.5) or *L. melissa* (c<0.5) ancestry, with enrichments of both types of excesses on the Z chromosome (randomization tests, 1000 randomizations each; c>0.5, Z observed = 23, Z expected = 16.4, *p* = 0.022; c<0.5, Z observed = 37, Z expected = 23.8, *p* = 0.001).

In general, variability in introgression among loci can reflect the joint effects of selection and genetic drift. A role for selection predicts associations between cline parameters and genomic features, such as chromosome size and genomic content (e.g., Schumer et al. 2018; Chaturvedi et al. 2020). Along these lines, we found a modest and marginally significant positive association between chromosome size and mean log cline slope or gradient (v) when considering the subset of chromosomes with at least five ancestry informative SNPs (linear regression, df = 17, β = 2.6 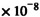, s.e. = 1.4 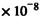, $r^{2}$ = 0.18, model *p* = 0.017). This would be expected if loci on larger chromosomes were affected on average more by indirect selection because of a lower rate of recombination per base pair (and thus higher average linkage disequilibrium). We found no evidence of steeper clines in or near (within 1 kb) genes (randomization test, 1000 randomizations, *p* = 0.792) but did find evidence of significantly steeper clines in or near annotated transposable elements (randomization test, 1000 randomizations, *p* = 0.011). Together these results suggest some role for selection in clinal patterns and highlight different patterns of introgression for autosomes and the Z chromosome. We followed up on this latter possibility with formal analyses comparing these sets of chromosomes.

In cline models based on autosomal hybrid indexes, we found credibly steeper clines on average for the Z chromosome than for autosomes (posterior probability $\mu_{v}$ for Z was greater than $\mu_{v}$ = 0.999 for autosomes, estimate of difference = 0.118, 90% CIs = 0.055–0.181; Figure 6H). This is consistent with stronger selection (or reduced recombination) in hybrids on the Z chromosome, especially as drift has a much more pronounced effect on cline centers than slopes in the absence of spatial structure (Gompert, Parchman, and Buerkle 2012). When considering cline slopes inferred from fully independent analyses of autosomes and Z loci (for hybrid indexes and clines), we found a trend towards more variability of introgression on the Z relative to average introgression on the Z ($\sigma_{v}$ = 0.355, 90% CI = 0.296–0.415) versus variability of introgression on the autosomes relative to average introgression on autosomes ($\sigma_{v}$ = 0.325, 90% CI = 0.273–0.374), but there was sufficient uncertainty in both parameters to preclude strong confidence in the difference suggested by this trend (posterior probability Z > autosomes = 0.748, see Figure 6I). Still, taken together, these results point to a special role for the Z sex chromosome in speciation in *Lycaeides* butterflies (consistent with Chaturvedi et al. 2020).

## 4 Discussion

Genomic analyses of hybrid zones provide unique and powerful insights into the nature and basis of species boundaries and the ecological and evolutionary consequences of hybridization (Harrison and Larson 2014; Gompert, Mandeville, and Buerkle 2017). Here, we described, demonstrated and assessed bgchm, a new R package designed to facilitate genomic analyses of hybrid zones. This R package combines methods and models for Bayesian inference hybrid indexes, ancestry class proportions, and genomic clines (and also geographic clines, see the Supporting Information, Table S9 and Figure S3) with HMC. We showed that bgchm provides accurate estimates of the relevant model parameters under a variety of conditions and especially when the genetic loci are highly informative of ancestry (i.e., when the allele frequency differences between source populations are not too small). This even includes reasonably robust estimates of the variability of clines across the genome via inference of cline standard deviations, which have not been the focus of previous models and methods. The models presented also allow for inference with uncertainty in genotypes, and we showed that at least with modest sequencing coverage this has minimal effect on the accuracy of inferences. Finally, we found that under most conditions true uncertainty in parameters was accurately estimated, although in some cases credible intervals were overly conservative (e.g., hybrid indexes with fixed differences between parents) or too narrow (e.g., genomic cline parameters in some cases).

Our results from simulated and empirical data sets build on our existing understanding of how evolutionary processes interact to affect patterns of introgression in hybrid zones (e.g., Endler 1977; Barton and Hewitt 1985; Gompert, Parchman, and Buerkle 2012; Harrison and Larson 2016; Gompert, Mandeville, and Buerkle 2017; McFarlane et al. 2021). For example, when hybrid fitness has a simple genetic architecture, loci residing in genomic regions proximate to causal variants affecting hybrid fitness had exceptional genomic cline parameters, consistent with Gompert, Parchman, and Buerkle (2012). The effects of selection on individual genomic cline parameters were less pronounced for weaker and more polygenic selection, though some signals remained in terms of cline parameters varying as a function of distance from causal variants in simulations and differences among classes of loci (those near transposable elements or on the Z sex chromosome versus autosomes) for the *Lycaeides* hybrid zone. This suggests that when the genetic architecture of hybrid fitness is polygenic, it is probably more informative to focus on such higher level contrasts, including cline standard deviations (which can sometimes be related to cline coupling, see, e.g., Firneno et al. 2023) rather than so‐called individual outlier loci as patterns of introgression for neutral and non‐neutral loci can be similar. It is also critical to recall that selection is not required for introgression to vary across the genome and for loci to deviate from null patterns of introgression based on genome‐wide admixture, as illustrated by our simulations of neutral secondary contact. Indeed, selection can either increase or decrease the variation in introgression across the genome, with the former expected for simple genetic architectures and the latter expected for coupled clines when many loci contribute to reproductive isolation (Barton 1983; Firneno et al. 2023). Thus, additional information beyond deviations from genome‐average introgression is required to infer processes from patterns in hybrid zones; we expand on this topic in the section 4.2.

### 4.1 Comparison With Other Software

Several computer programs exist for genetic analyses of hybrid zones, and thus, it is worth considering how this newly introduced R package, bgchm, fits in with existing software. To our knowledge, four main programs are currently available for estimating genomic clines. The earliest of these was introgress (Gompert and Buerkle 2010), which adopts a multinomial likelihood‐based approach to estimate genomic clines for multilocus genotypic data (Gompert and Buerkle 2009, 2010). This program does not consider ancestry but is unique in separately modeling introgression of homozygous versus heterozygous genotypes. The original bgc (Gompert and Buerkle 2012) fits Bayesian genomic clines in ancestry using a hierarchical model and a polynomial function for clines adapted from Szymura and Barton's (1986). This software has many similarities with our new bgchm, including the basic hierarchical modeling approach and the ability to work with genotype uncertainty. However, bgc is less modular (all loci must be fit together) and uses traditional Markov chain Monte Carlo, which exhibits notably poorer mixing. These features make bgc less well‐suited for genome‐scale data and for estimating cline standard deviations (these tend to mix especially poorly and are generally treated as nuisance parameters). HIest features multiple cline models and approaches to model fitting but takes a non‐hierarchical approach and assumes fixed differences between source populations (Fitzpatrick 2012). Finally, the recently released gghybrid (Bailey 2024) shares many aspects with bgchm including the Bayesian approach and use of the logit‐logistic cline model. The key features provided by bgchm relative to gghybrids are (i) an ability to model uncertainty in genotypes, (ii) the ability to directly model ancestry data, (iii) the use of hierarchical models and thus inference of cline standard deviation, (iv) interpopulation ancestry models, (v) hierarchical Bayesian models for geographic cline analyses, and (vi) the use of HMC for efficient sampling from posterior distributions. In terms of speed, the original bgc is by far the slowest program, especially with large data sets, whereas the likelihood‐based approaches tend to be the fastest. We have not conducted a detailed comparison of gghybrid and bgchm, and this is slightly complicated by the fact that fewer MCMC steps are required to obtain a high effective sample size with HMC, but both programs make it practical to analyze very large data sets, especially given the potential for parallelization in bgchm (and gghybrids). For bgchm, the total runtime largely depends on the extent to which cline fitting is done in parallel after the cline standard deviation parameters have been estimated. With robust computational resources (i.e., a single compute node with ~48 CPUs and multi‐threading), we have been able to successfully fit clines for millions of SNPs in a few days of human time.

Further, existing programs differ in terms of the set of features included. The original bgc was a standalone program that only included the genomic cline model but did estimate hybrid indexes as part of this model. In contrast, introgress, HIest, and gghybrid include additional functions for estimating hybrid indexes and (for the former two) for estimating genotype‐based metrics similar to ancestry class proportions. bgchm also includes models of hybrid index inference and includes a unique model for true ancestry class proportions (this is similar to the Q model in entropy, but with source populations designated a priori; Gompert et al. 2014; Shastry et al. 2021). Additionally, while several computer programs, including Cfit (Gay et al. 2008) and hzar, which uses a Bayesian approach (Derryberry et al. 2014), exist for inference of geographic cline parameters, bgchm is unique in including the option to fit hierarchical models for geographic and genomic clines in a single program (the geographic cline models are described in the Supporting Information). Likelihood‐based approaches for estimating hybrid index and interpopulation ancestry exist in introgress (Gompert and Buerkle 2010) and HIest (Fitzpatrick 2012), and hybrid indexes can be inferred in several programs using either likelihood or Bayesian methods (e.g., introgress, HIest, bgc, and gghybrid; Gompert and Buerkle 2012; Bailey 2024). Moreover, interpopulation ancestry and admixture proportions, which are analogous to hybrid indexes with two source populations, can be jointly inferred in entropy (Gompert et al. 2014; Shastry et al. 2021). Finally, additional software packages exist for genomic analyses of hybrids or hybrid zones that focus on ancestry inference via genome polarization without pre‐defined parental populations (Baird et al. 2023) and genomic characterization of hybrids from diagnostic markers (Wiens and Colella 2024).

### 4.2 Conclusions and Future Directions

Our use of HMC, and specifically the NUTS algorithm from Stan, results in more rapid and robust Bayesian inference of genomic clines than was possible with the original bgc program. However, analyses of very large data sets, or of many replicate hybrid zones, can still require substantial time or computational resources (e.g., many CPUs). One possible way to overcome this limitation is to replace the current HMC approach with an approximation of the posterior through variational inference (Kucukelbir et al. 2017). Variational inference is supported by Stan and allows for automatic approximation of the posterior distribution. This can increase the speed of model fitting by orders of magnitude (Kucukelbir et al. 2017). However, it can also come at a cost in terms of accuracy, and the reliability of variational inference for genomic cline models remains to be evaluated. We see additional potential for increases in speed, and potentially accuracy, by fitting cline models for ancestry blocks (as identified in models for local ancestry inference or via genome polarization, e.g., Sankararaman et al. 2008; Baird et al. 2023; Browning, Waples, and Browning 2023) rather than for genotypes or ancestry at individual loci. This could reduce the number of independent genomic regions or loci required for analysis and simultaneously overcome limitations that arise from low ancestry information for subsets of loci. This could be done with the existing ancestry model in bgchm. We intend to evaluate both variational approximations and ancestry‐block based analyses in a future publication.

Finally, hybrid indexes, ancestry class proportions, and genomic clines provide summaries of patterns of introgression but connecting such genomic patterns to ecological and evolutionary processes remains difficult (McFarlane et al. 2021). With certain assumptions or information, especially about dispersal, geographic patterns of introgression can be directly related to process‐based parameters, such as the average intensity of selection against hybrids (e.g., Barton and Hewitt 1985; Szymura and Barton 1986; Mallet et al. 1990). However, this is less true for genomic clines, as these are always relative to overall admixture and thus not absolute metrics of introgression (this is also an advantage as they are less dependent on the geography of a hybrid zone). We think a valuable area for future research is to test whether the combined information from hybrid indexes, ancestry class proportions, genomic and geographic clines, as well as patterns of linkage disequilibrium in hybrid zones, could be used to reliably infer demographic and evolutionary processes governing hybrid zones, at least for a subset of clear, alternative models. This could be done using approximate Bayesian computation or with neural networks, both of which are suitable for combining information across heterogeneous data types (Sisson, Fan, and Beaumont 2018; Gehara, Mazzochinni, and Burbrink 2020; Yang et al. 2022). Convolutional neural networks, which have recently shown great general promise in population genomics (Flagel, Brandvain, and Schrider 2019; Torada et al. 2019; Smith et al. 2023), could be particularly useful for mapping such disparate data information sources to generative processes that emit identifiable signals. We think that this gap between pattern and process is an important area for future work to address and we hope to contribute to doing so in future work.

## Author Contributions

**Zachariah Gompert:** conceptualization (lead), formal analysis (lead), funding acquisition (lead), investigation (lead), methodology (lead), software (lead), writing – original draft (lead), writing – review and editing (lead). **Devon A. DeRaad:** software (supporting), writing – review and editing (equal). **C. Alex Buerkle:** conceptualization (equal), software (equal), writing – review and editing (equal).

## Conflicts of Interest

The authors declare no conflicts of interest.

## Code Availability

The source code for bgchm can be downloaded and installed from GitHub (https://github.com/zgompert/bgc‐hm). Additional scripts used for simulations and analyses in this article are available from a second GitHub repository (https://github.com/zgompert/bgchm_test).

## Supporting information


Data S1.

## Data Availability

Simulated data sets are available from Dryad (https://doi.org/10.5061/dryad.tht76hf87). DNA sequence data for the butterfly hybrid zone are available from the NCBI SRA (PRJNA577236 and PRJNA432816).
