# Supplementary material for: A Next Generation of Hierarchical Bayesian Analyses of Hybrid Zones Enables Model‐Based Quantification of Variation in Introgression in R
Source: Ecol Evol. 2024 Nov 21;14(11):e70548. doi: 10.1002/ece3.70548 (PMC11582016; doi:10.1002/ece3.70548)
Supplement: Supplementary file 1 — Data S1. [file ECE3-14-e70548-s001.pdf]

Supplemental Methods and Results

Geographic cline model

A variety of hybrid zone models predict sigmoidal allele frequency clines such that $p = [1 + \tanh\left(\frac{2(x_j - c)}{w}\right)]/2$, where w is the cline width, c is the cline center, and x is the location of deme j (e.g., Szymura & Barton, 1991; Barton *et al.*, 1993). This includes cases where clines are neutral (e.g., result from recent secondary contact and are not maintained by selection) and cases where clines are maintained by the opposing processes of dispersal and various forms of endogenous or exogenous selection (Barton & Hewitt, 1985; Barton *et al.*, 1993). When many loci contribute to hybrid fitness, clines can be coupled such that each experiences, indirectly (via linkage disequilibrium), some of the selection caused by the effects of the other loci (this applies even to neutral loci) (Barton, 1983). When this occurs, clines are expected to retain the sigmoidal form in the center of the hybrid zone (with a width dictated by direct and indirect selection), but exhibit a distinct pattern of exponential decay in allele frequency away from the hybrid zone center (Barton, 1983). Thus, the sigmoidal function applies to both single locus and multi-locus clines, but only away from the edges of the hybrid zone. This central, sigmoidal, portion of the hybrid zone can be described by a linear function on a logit scale, that is $\text{logit}(p_{ij}) = c_i + \beta_i x_j$ (Szymura & Barton, 1991; Barton *et al.*, 1993). Here, c_i and β_i are the center (intercept on the logit scale) and slope of the cline for locus i and x_j is the (centered) geographic coordinate for deme j. The cline width on the natural scale (w_i) is related to the slope as $w_i = \frac{4}{\beta_i}$. We work with this logit-linear geographic cline function.

We specifically assume the logit-linear function above describes the allele frequency cline over some geographic region, specifically the geographic region where allele frequencies vary between a defined minimum and maximum value (i.e., away from the edges of the cline or hybrid zone). In `bgchm`, this value can be set by the user, but we use -2 to 2 on the logit scale by default, ~0.12 to ~0.88 on the natural scale (as in Kruuk *et al.*, 1999). We thus assume that the likelihood of $\text{logit}(p_{ij})$ (logit allele frequency for locus i and population j) is $\text{logit}(p_{ij}) \sim \text{normal}(c_i + \beta_i x_j, \sigma_\epsilon)$. In this model σ_ϵ is a standard deviation parameter describing the residual error.

Our main interest is in estimating the slopes (and associated cline widths). We do this using a hierarchical model, which also allows us to estimate the variability in slopes (and thus cline widths) across the genome (see the main text for a discussion of the general benefits of hierarchical modelling). We thus place a normal prior on the slopes, $\beta_i \sim \text{normal}(\mu_\beta, \sigma_\beta)$, where μ_β and σ_β describe the mean and standard deviation of slopes and are estimated from the data (this is a hierarchical prior). In contrast, we use a simple, relatively uninformative, non-hierarchical prior for the centers, $c_i \sim \text{normal}(0, \sigma_0)$. We set the mean to 0 as the geographic coordinates are centered. We set σ_0 based on the scale of the geographic coordinates, specifically, $\sigma_0 = 3\text{SD}(x)$, where $\text{SD}(x)$ is the standard deviation of the geographic coordinates. We complete the model by specifying priors, which by default are relatively uninformative, for the remaining parameters: $\mu_\beta \sim \text{normal}(0, \sigma_\mu)$, $\sigma_\beta \sim \text{gamma}(\alpha = \alpha_0, \beta = \beta_0)$, and $\sigma_\epsilon \sim \text{gamma}(\alpha = \alpha_0, \beta = \beta_0)$. Here, α_0 and β_0 are set by the users (the current defaults

are small values, 0.1 and 0.01), and σ_μ is set relative to the scale of the geographic coordinates, $\sigma_\mu = 1.5\frac{4}{\mathrm{SD}(x)}$. As with the genomic cline models, the geographic cline models are fit using HMC with the NUTS algorithm via Stan (Neal *et al.*, 2011; Betancourt & Girolami, 2015; Stan Development Team, 2022, 2024). This is done with the `est_geocl` function in `bgchm`.

Simulations and results for geographic clines

We analyzed a series of simulated data sets to validate the performance of `bgchm`'s geographic clines model, with emphasis on (i) the effect of cline variability on inference, and (ii) our ability to estimate cline variability. Similar to our initial tests of the genomic clines models, we simulated data using the geographic clines model as our generative model. This has the key strength of providing known parameter values that can be compared to our estimates. We simulated three levels of cline variability, that is standard deviations in slopes (on the logit-linear scale) among loci (across the genome) of $\sigma_\beta = 0.1$, 0.4 and 0.8. We set the standard deviation for centers (again on the logit-linear scale) to 0.3 and the residual error standard deviation (σ_ϵ) to 0.1. We simulated 100 data sets, each comprising 25 demes and 100 loci, under each of these three levels of cline variability.

We first sampled geographic coordinates for the 25 demes from a standard normal distribution. The resulting coordinates were centered (i.e., forced to have a mean of zero). Next, for each data set, we sampled cline centers and slopes for the 100 loci from normal distributions, $\beta_i \sim \mathrm{normal}(\mu_\beta, \sigma_\beta)$ and $c_i \sim \mathrm{normal}(0, 0.3)$. Here, μ_β is the mean slope across loci, which we set to 1.75. We then calculated expected logit allele frequencies for each deme and locus as $\mathrm{logit}(p_{ij}) = c_i + \beta_i x_j$; actual logit allele frequencies were sampled from a normal distribution centered on this value but with a standard deviation of σ_ϵ. The resulting allele frequencies (converted to the natural scale), were used as input for `bgchm` along with the geographic coordinates. We estimated geographic cline parameters with the `est_geocl` function using the default HMC settings of four chains, 2000 total iterations, a 1000 iteration warmup, and a thinning interval of 1.

We found that `bgchm` produces remarkably accurate and precise estimates of cline parameters, including the standard deviation parameter, which is perhaps unsurprising given the general ease of inference for (hierarchical) linear models (Table S9 and Figure S3). Specifically, the slope standard deviation estimates corresponded closely with the true values of 0.1 (mean across simulations = 0.099), 0.4 (mean = 0.395) and 0.8 (mean = 0.789) (Figure S3B). Moreover, mean absolute errors for slopes and centers for individual loci were uniformly low (means < 0.037 and 0.019, respectively) and correlations between true and estimated parameter values were high (mean correlations ranged from 0.925 to 0.998) (Table S9 and Figure S3C-F). Finally, 90% CIs for these parameters contained the true values about the expected proportion of the time, that is about 90% of the time (Table S9)

Supplemental Tables and Figures

Table S1: Mean absolute error (MAE) and 90% credible interval coverage (90% CI cov.) for hybrid index and interpopulation ancestry parameters from `bgchm` summarized across 50 replicate simulations for each of six sets of conditions. Conditions considered were allele frequency differences (AFDs) of 1, 0.5 or 0.1 with known or uncertain genotypes. Means and standard deviations (in parentheses) across the 50 replicates are given for each metric and set of conditions.

	Hybrid index (H)		Interpopulation ancestry (Q_{10})	
Conditions	MAE	90% CI cov.	MAE	90% CI cov.
AFD 1.0, known genotypes	0.002 (0.0002)	1.000 (0.000)	0.004 (0.0006)	1.000 (0.00)
AFD 0.5, known genotypes	0.021 (0.0027)	0.944 (0.031)	0.063 (0.0071)	0.910 (0.039)
AFD 0.1, known genotypes	0.130 (0.0116)	0.889 (0.040)	0.161 (0.0161)	0.976 (0.019)
AFD 1.0, uncertain genotypes	0.004 (0.0005)	1.000 (0.000)	0.008 (0.0010)	1.000 (0.003)
AFD 0.5, uncertain genotypes	0.022 (0.0025)	0.937 (0.034)	0.069 (0.0082)	0.910 (0.041)
AFD 0.1, uncertain genotypes	0.133 (0.0134)	0.889 (0.051)	0.166 (0.0196)	0.977 (0.021)

Table S2: Mean absolute error (MAE) and correlations between true and estimated parameter values for genomic cline slope (v) and center (c) parameters. Results are shown for different cline standard deviations (σ_v or σ_c) and with inferences based on a non-hierarchical model from `bgchm` (`bgchm`-nh), the standard hierarchical model from `bgchm` (`bgchm`-hm), or `HIest`. Means across 50 replicate simulations with fixed allele frequency differences between parents are shown.

		MAE			Correlation		
Parameter	σ	`bgchm`-nh	`bgchm`-hm	`HIest`	`bgchm`-nh	`bgchm`-hm	`HIest`
v	0.2	0.230	0.189	0.221	0.874	0.882	0.875
v	0.4	0.367	0.337	1.461	0.912	0.917	0.777
v	0.6	0.966	0.972	8.225	0.781	0.789	0.640
c	0.5	0.050	0.044	0.052	0.864	0.877	0.854
c	0.8	0.055	0.052	0.064	0.901	0.910	0.860
c	1.2	0.060	0.061	0.078	0.915	0.919	0.853

Table S3: Proportion of loci with 90% credible intervals (CIs) overlapping the true value or 0 (the null expectation) for genomic cline slope (v) and center (c) parameters. Results are shown for different cline standard deviations (σ_v or σ_c) and with inferences based on a non-hierarchical model from `bgchm` (`bgchm`-nh) or the standard hierarchical model from `bgchm` (`bgchm`-hm). Means across 50 replicate simulations with fixed allele frequency differences between parents are shown.

		CI overlap true value		CI overlap 0	
Parameter	σ	`bgchm`-nh	`bgchm`-hm	`bgchm`-nh	`bgchm`-hm
v	0.2	0.89	0.89	0.53	0.58
v	0.4	0.88	0.87	0.30	0.31
v	0.6	0.81	0.78	0.21	0.22
c	0.5	0.90	0.87	0.55	0.61
c	0.8	0.89	0.87	0.43	0.46
c	1.2	0.90	0.84	0.36	0.38

Table S4: Mean absolute error (MAE), correlations between true and estimated parameter values, and proportion of loci with 90% credible intervals (CIs) overlapping the true value for genomic cline slope (v) and center (c) parameters. Conditions considered were minimum allele frequency differences (AFDs) of 1, 0.5 or 0.1 with known or uncertain genotypes. Means across 50 replicates for each set of simulation conditions are shown.

	MAE		Correlation		CI overlap 0	
Conditions	v	c	v	c	v	c
AFD 1.0, known genotypes	0.24	0.05	0.93	0.91	0.88	0.87
AFD 0.5, known genotypes	0.43	0.07	0.77	0.85	0.87	0.86
AFD 0.1, known genotypes	0.68	0.09	0.60	0.71	0.87	0.81
AFD 1.0, uncertain genotypes	0.26	0.05	0.92	0.90	0.88	0.88
AFD 0.5, uncertain genotypes	0.45	0.07	0.76	0.84	0.87	0.85
AFD 0.1, uncertain genotypes	0.71	0.09	0.58	0.69	0.87	0.80

Table S5: Numbers of loci with credible deviations from genome-average introgression based on 90% equal-tail probability intervals. Results are summarized for ten replicates each of neutral, oligogenic (two underdominant loci), and weak and strong polygenic (50 underdominant loci) selection and for cline gradient (v) and center (c). Means and standard deviation s (SDs) across the ten replicates are reported; numbers are out of 251 loci.

	gradient (v)		center (c)	
Conditions	mean	SD	mean	SD
Neutral	74.2	4.4	184.4	3.9
Oligogenic	68.5	5.6	175.9	8.7
Weak polygenic	74.6	5.2	184.9	5.7
Strong polygenic	68.7	6.5	168.2	7.4

Table S6: Correlation between point estimates of log cline gradient parameters (v) and the distance in centimorgans to the nearest underdominant locus. Results are summarized for ten replicates each of neutral, oligogenic (two underdominant loci), and weak and strong polygenic (50 underdominant loci) selection. For neutral simulations, there were not underdominant loci, and thus we used the underdominant locus positions from the polygenic simulations (this is done to verify observed patterns with selection are not artifactual). Cases where the reported correlation is significantly different from 0 ($P < 0.05$) are in bold.

Replicate	Neutral	Oligogenic	Weak polygenic	Strong polygenic
1	0.05	**-0.36**	-0.07	**-0.16**
2	0.04	**-0.47**	-0.11	**-0.19**
3	0.04	**-0.48**	-0.08	**-0.11**
4	0.00	**-0.37**	-0.08	**-0.14**
5	0.06	**-0.48**	-0.07	**-0.20**
6	-0.02	**-0.48**	-0.10	**-0.16**
7	0.05	**-0.42**	-0.06	**-0.13**
8	0.01	**-0.49**	-0.09	**-0.18**
9	0.03	**-0.45**	-0.04	**-0.13**
10	0.03	**-0.34**	-0.08	**-0.23**

Table S7: Correlation between point estimates of the absolute value of logit cline gradient centers (c) and the distance in centimorgans to the nearest underdominant locus. Results are summarized for ten replicates each of neutral, oligogenic (two underdominant loci), and weak and strong polygenic (50 underdominant loci) selection. For neutral simulations, there were not underdominant loci, and thus we used the underdominant locus positions from the polygenic simulations (this is done to verify observed patterns with selection are not artifactual). Cases where the reported correlation is significantly different from 0 ($P < 0.05$) are in bold.

Replicate	Neutral	Oligogenic	Weak polygenic	Strong polygenic
1	0.03	**0.20**	0.04	**0.18**
2	0.01	**0.28**	0.07	**0.17**
3	-0.01	**0.25**	0.05	0.08
4	-0.02	**0.15**	0.07	**0.13**
5	0.01	**0.25**	0.05	**0.15**
6	0.02	**0.29**	0.10	**0.13**
7	0.03	**0.29**	0.05	0.11
8	0.01	**0.19**	0.03	**0.13**
9	-0.01	**0.17**	0.09	**0.13**
10	0.02	**0.28**	0.03	**0.17**

Table S8: Summary of evidence for an excess of loci deviating from genome-wide introgression on the Z chromosome. Deviations considered include $v > 1$ (steeper clines), $v < 1$ (shallower clines), center > 0.5 (excess Jackson Hole *Lycaeides* ancestry) and center < 0.5 (excess *L. melissa* ancestry). Classifications are based on the 90% credible intervals for each parameter excluding the relevant null value (1 or 0.5) in the specified direction. The total number of such loci, the number on the Z, and the number on the Z expected by chance given the number of autosomal and Z SNPs analyzed (based on the mean of 1000 randomizations) are given, along with the associated P-value for the number on the Z exceeding the null expectations from the 1000 randomizations.

Condition	Total	Z observed	Z expected	P-value
$v > 1$	40	39	13.7	0.001
$v < 1$	60	12	40.5	0.999
center > 0.5	48	23	16.4	0.022
center < 0.5	70	37	23.8	0.001

Table S9: Mean absolute error (MAE), correlations between true and estimated parameter values, and proportion of loci with 90% credible intervals (CIs) overlapping the true value for geographic cline slope (β) and center (c) parameters. Results are shown for slope standard deviations of 0.1, 0.4 and 0.8 Means across 100 replicates for each set of simulation conditions are shown.

	MAE		Correlation		CI overlap 0	
σ	β	c	β	c	β	c
0.1	0.031	0.017	0.925	0.997	0.889	0.904
0.4	0.034	0.018	0.994	0.997	0.893	0.904
0.8	0.036	0.018	0.998	0.997	0.891	0.901

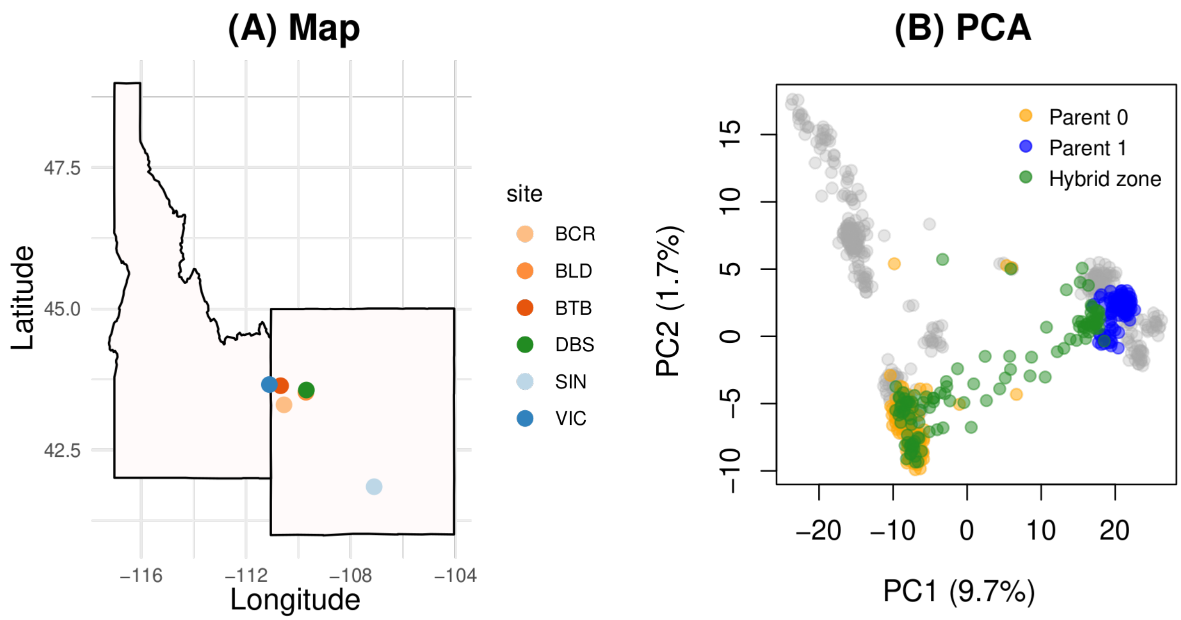

Figure S1: Map and summary of genetic variation for the *Lycaeides* hybrid zone. The map shows outlines for the US states of Idaho and Wyoming along with points for the focal *Lycaeides* populations in this paper (A). This includes the two *L. melissa* populations (SIN and VIC), the three Jackson Hole *Lycaeides* populations (BCR, BLD and BTB), and the hybrid zone population (DBS). Panel (B) summarizes patterns of genetic variation with a principal components analysis (PCA). The first two PC axes are shown. Each point denotes an individual butterfly. Orange and blue points correspond with Jackson Hole *Lycaeides* and *L. melissa* reference source populations, respectively. Green points denote individuals form the Dubois hybrid zone. Gray dots indicate butterflies not included in the hybrid zone analysis, including non-admixed *L. idas*.

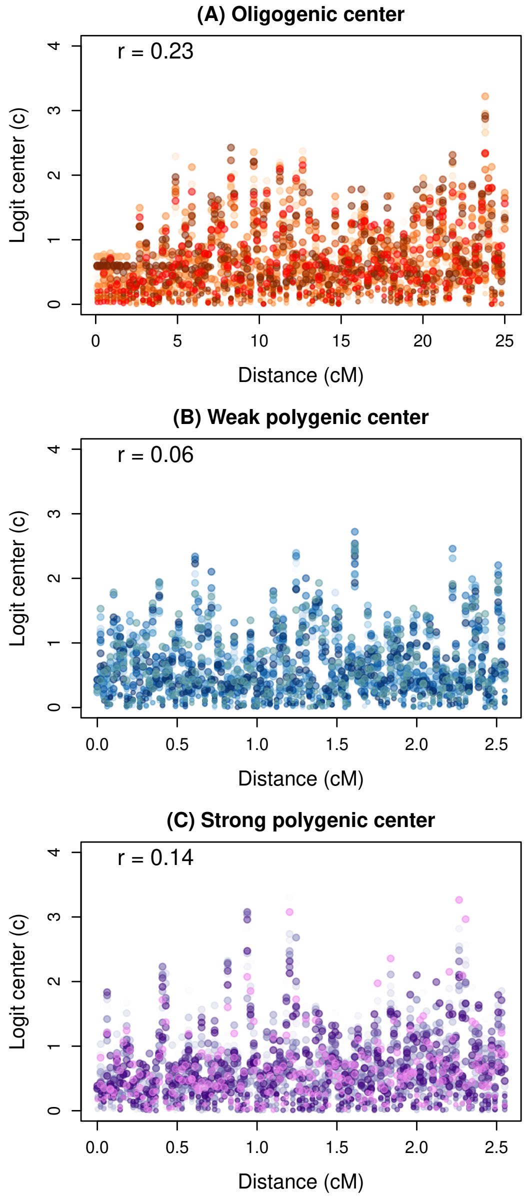

Figure S2: Summary of genomic cline center estimates for hybrid zone simulations with alternative genetic architectures for hybrid fitness. Results are shown for oligenic selection (A), weak polygenic selection (B) and strong polygenic selections (C) (see main text for details). Plots show the relationships between the distance (in cM) a marker locus is from a selected locus and the logit of the cline center (c). This is only shown for the three sets of conditions with selection. Points are colored to indicate distinct replicate simulations and the Pearson correlation between distance and logit(c) is reported.

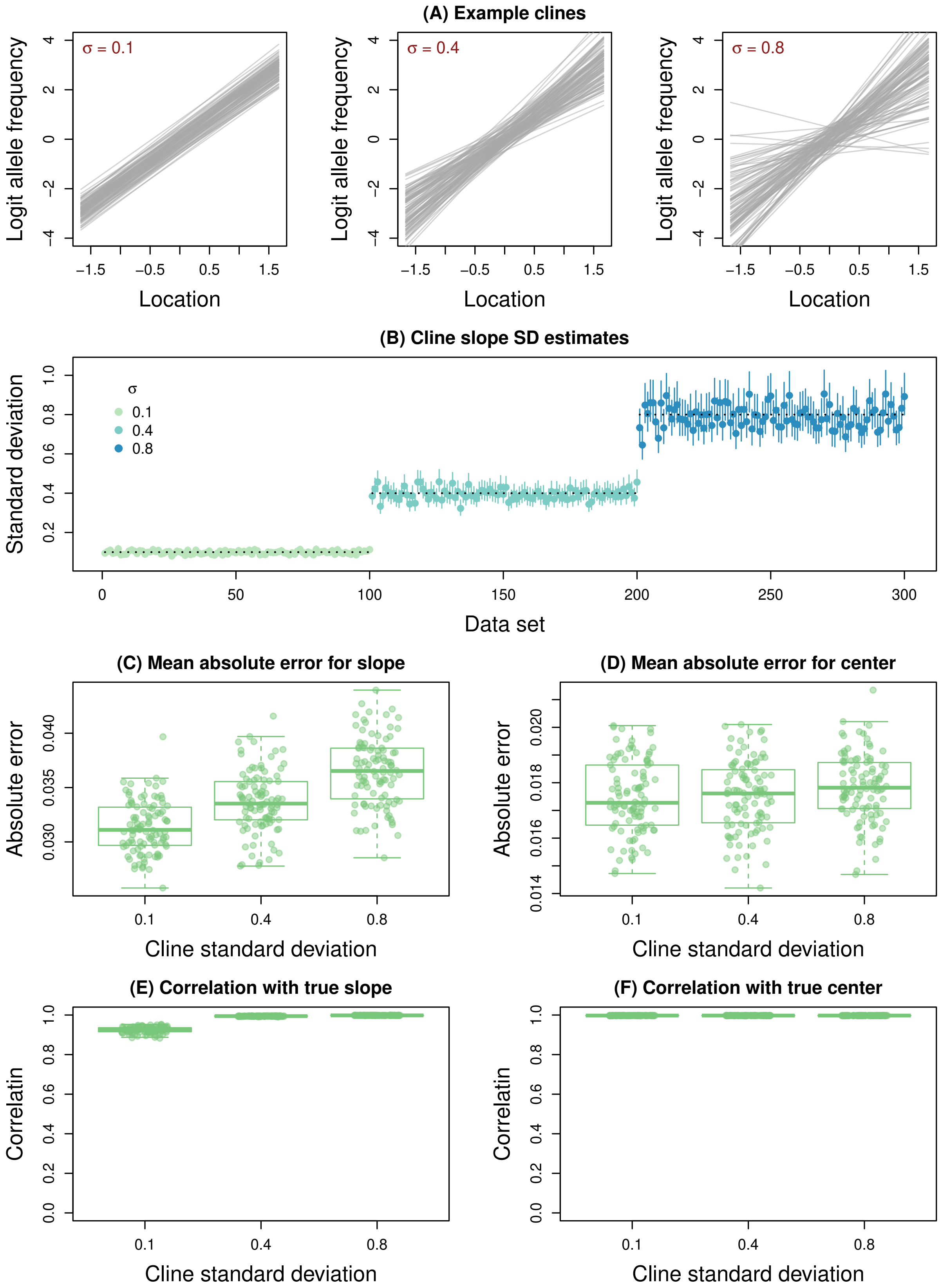
(A) Example clines
σ = 0.1
σ = 0.4
σ = 0.8
Logit allele frequency
Location
(B) Cline slope SD estimates
σ
0.1
0.4
0.8
Standard deviation
Data set
(C) Mean absolute error for slope
(D) Mean absolute error for center
Absolute error
Cline standard deviation
(E) Correlation with true slope
(F) Correlation with true center
Correlatin
Cline standard deviation

Figure S3: Summary of geographic cline variability and the effect of such variability on cline inference. Panel (A) shows geographic cline estimates with low ($\sigma = 0.1$), moderate ($\sigma = 0.4$), and high ($\sigma = 0.8$) variability in slopes on the logit scale. Each gray line is the cline for a locus and gives the expected logit allele frequency as a function of geographic location. Estimates of cline standard deviations for slopes are shown in panels (B). Here, point estimates and 90% credible intervals (CIs) are depicted with points and vertical lines. Horizontal dotted lines give the true value used for each simulation. Performance, in terms of estimating cline slopes (β) and centers, is summarized based on mean absolute error in panels (C) and (D) and in terms of the correlation between true and estimated parameter values in panels (E) and (F). Errors and Pearson correlations were computed based on parameter point estimates (posterior medians) and are summarized across replicate simulations with boxplots. Boxes indicate the median and 1st and 3rd quartiles of the distribution across replicate simulations, with whiskers extending up to $1.5\times$ the interquartile range. The overlain points show metrics for individual replicates.
